# Depolymerization of PLA catalyzed by guanidine-modified microgels

**DOI:** 10.1039/d5sc03443d

**Published:** 2025-09-23

**Authors:** Fabian Fink, Frédéric Grabowski, Sandra Oden, Paul Nisgutski, Andrij Pich, Sonja Herres-Pawlis

**Affiliations:** a Institute of Inorganic Chemistry, RWTH Aachen University Aachen D-52074 Germany sonja.herres-pawlis@ac.rwth-aachen.de; b Institute of Technical and Macromolecular Chemistry, RWTH Aachen University Aachen D-52074 Germany; c DWI – Leibniz Institute for Interactive Materials Aachen D-52074 Germany pich@dwi.rwth-aachen.de; d Aachen Maastricht Institute for Biobased Materials, Maastricht University RD Geleen 6167 The Netherlands

## Abstract

Microgels are smart catalyst carrier systems, providing good catalyst solubility, accessibility, and compartmentalization, enabling recyclability and improving catalytic performance. In this study, we synthesized functional microgels with controlled number and localization of guanidine units. The microgels were evaluated for their catalytic performance in the methanolysis of polylactide (PLA) and demonstrated high catalytic performance of the guanidine catalyst. In addition, the localization of guanidine in the microgel core led to a faster depolymerization of PLA. The microgel carrier reduced the deactivation of the guanidine units compared to the unsupported guanidine catalyst over several cycles. Our results demonstrate the potential of microgels as catalyst carrier systems that can be used in multiple reaction cycles to improve the implementation of a circular plastics economy and thereby help to reduce plastic pollution in the environment.

## Abstract 2

Guanidine-modified microgels were developed as organocatalyst carrier system. These microgels enable the depolymerization of polylactide by methanolysis and show remarkable recyclability over repeated cycles while maintaining catalytic performance.

## Graphical abstract

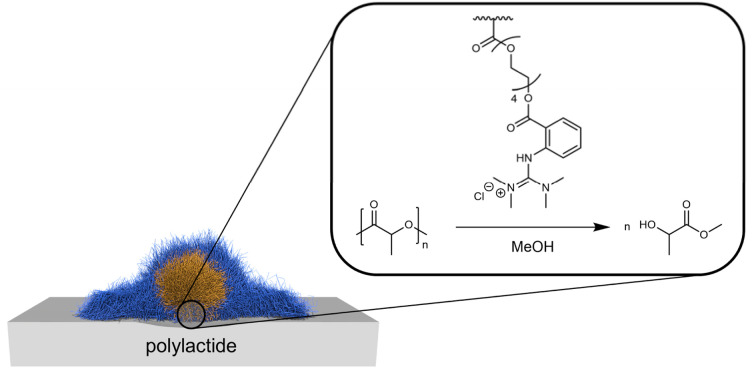

## Introduction

Plastics made from petrochemical feedstocks are an indispensable part of today's standard of living, due to their versatile and customized properties. On account of their cost-effective production and durability, plastic materials are used in nearly all areas of modern life.^1,2^ As a result of the thoughtless handling and incorrect disposal of non-degradable plastic waste, many marine and terrestrial ecosystems are being heavily polluted.^2,3^ Aiming to tackle the issue, bio-based and biodegradable plastics are being introduced into the plastics economy. Unfortunately, the effectiveness of these measures is not sufficient to prevent the accumulation of end-of-life (EoL) plastics.^4,5^ This requires more efficient strategies that consider a circular economy of plastics which are designed for recycling.^6–9^ By far the most utilized recycling method is mechanical recycling.^10,11^ For this, pure plastic wastes are very suitable, but the number of recycling cycles in this method is limited, and contamination by additives or other polymers and undesired side reactions also contribute to the deterioration of the material.^6,11^

Lately, the importance of chemical recycling has increased as a method of obtaining high-quality polymer materials. Here, polymer chains are broken down into smaller molecules such as monomers or platform chemicals that can be reused to prepare new polymers or other value-added materials.^10,12–14^ The method also allows for the degradation of plastic mixtures by selective degradation of a particular polymer type under suitable reaction conditions, *e.g.* polylactide (PLA).^10,13,15–17^ In general, polyesters are well suited for chemical recycling, because their ester bonds in the polymer chain are readily accessible by hydrolysis, alcoholysis, or aminolysis.^18–20^

PLA is a promising polyester that can be completely degraded under industrial composting conditions and is based on lactic acid.^21,22^ Thus, this polymer is a fully bio-based, biocompatible, and biodegradable aliphatic polyester^21,23–25^ and therefore a promising candidate for setting up a bio-based circular plastics economy with efficient EoL disposal options.^19,24,26–28^ In fact, PLA is already used as packaging material for food products, such as yogurt,^29^ even as disposable cutlery,^30^ as mulch film in agriculture,^31^ and in medical applications.^32–34^

In the industrial production of PLA, tin octanoate (Sn(Oct)_2_) is commonly used as a polymerization catalyst, which is cytotoxic and remains in small amounts in the product after polymerization.^35–37^ Since the plastics degrade, the cytotoxic Sn(Oct)_2_ can concentrate in the environment and pose a potential hazard.^36,37^ Therefore, research is being conducted on the replacement of the industrial catalyst with a more environmentally friendly, biocompatible catalyst. Various catalysts are already known that are more active than the industrially used tin catalyst.^26,38–40^ Furthermore, specific catalysts (*e.g.* Zn, Mg, and Fe complexes) are able to catalyze both the polymerization of lactide (LA) and the depolymerization of PLA.^8,15,26,27,41–44^ While many studies focus exclusively on the polymerization of LA, a limited number address the increasingly important depolymerization of PLA to support a more sustainable and circular plastics economy.

In this regard, a promising recycling method of PLA is the alcoholysis, as various alcohols can be converted into alkyl lactates, which can be used as green solvents or converted into LA.^45–47^ Among alcoholysis, methanolysis is the most frequently used.^24,41,48^ A number of non-toxic catalysts are available that are suitable for the alcoholysis of PLA, including metal complexes of Al,^49^ Ti,^49^ Bi,^50^ Mg,^51^ and Zn.^15,41,52^ Especially metal guanidine complexes of Fe^44,53^ and Zn^17,26,27,54^ show outstanding activities. Furthermore, there are various catalysts able to degrade PLA without the use of metals, like ionic liquids^55,56^ and also organocatalysts,^57–59^*e.g.* the bicyclic guanidine 1,5,7-triazabicyclo[4.4.0]dec-5-ene (TBD).^45,59–61^

However, guanidines are homogeneous catalysts that are challenging to separate and recycle.^62–64^ This drawback can be overcome by developing catalyst carriers designed to preserve catalytic activity while at the same time enhancing the recyclability. For this purpose, rigid colloids based on silica or polystyrene have been coated on the surface with guanidine-based catalysts and the developed solid catalyst carriers could thus be recycled in multiple cycles.^65,66^ Other approaches improve the recovery by immobilizing guanidine-based catalysts on magnetic nanoparticles.^67,68^ In addition, metal–organic frameworks (MOFs) are used on account of their large inner surface area and characteristic stability.^69^ The immobilization of all these carrier systems is based on non-responsive materials that only act passively. Furthermore, the number of catalytically active centers is only partially controllable, and the localization within these carrier systems cannot be controlled by any means. To tackle these concerns, stimuli-responsive microgels can be used as a catalyst carrier system so that the catalytic performance can be modulated by external modification and also allows different compartmentalization of the catalyst, which further affects the catalytic behavior.^70–75^ In addition, poly(*N*-vinylcaprolactam) (PVCL) based microgels are biocompatible and biodegradable, making them suitable as a sustainable carrier system material.^76,77^

Herein, we report on the compartmentalization of various contents and localizations of guanidine units in microgels to obtain guanidine-modified microgels. For this purpose, a water-soluble guanidine-modified monomer based on tetraethylene glycol (TEG) was developed. The synthesized protonated tetramethyl guanidine tetraethylene glycol methacrylate (TMGtegma+) monomer was then incorporated as comonomer in PVCL-based microgels with different amounts (5, 10, and 15 mol%) *via* batch and semi-batch precipitation polymerization. The prepared microgels were investigated for their swelling behavior using dynamic light scattering (DLS) and the particle dispersity together with the morphology was analyzed by bright-field scanning transmission electron microscopy (BFSTEM) images. Nuclear magnetic resonance (NMR) spectroscopy was used to determine the successful incorporation and the comonomer content in the microgels. Subsequently, the microgels were screened for their catalytic performance in the methanolysis of PLA. The influence of temperature, time, and the stabilization properties of the microgel carrier systems for the guanidine catalysts were evaluated under inert and air atmosphere. In the final step, the methanolysis was repeated in multiple cycles, demonstrating the recyclability of the microgel carrier systems. This illustrates the advancing development towards a circular economy in which microgel carrier systems facilitate easy removal and recyclability of catalysts for several catalytic cycles.

## Results

### Synthesis and characterization of guanidine modified microgels

**Fig. 1:**
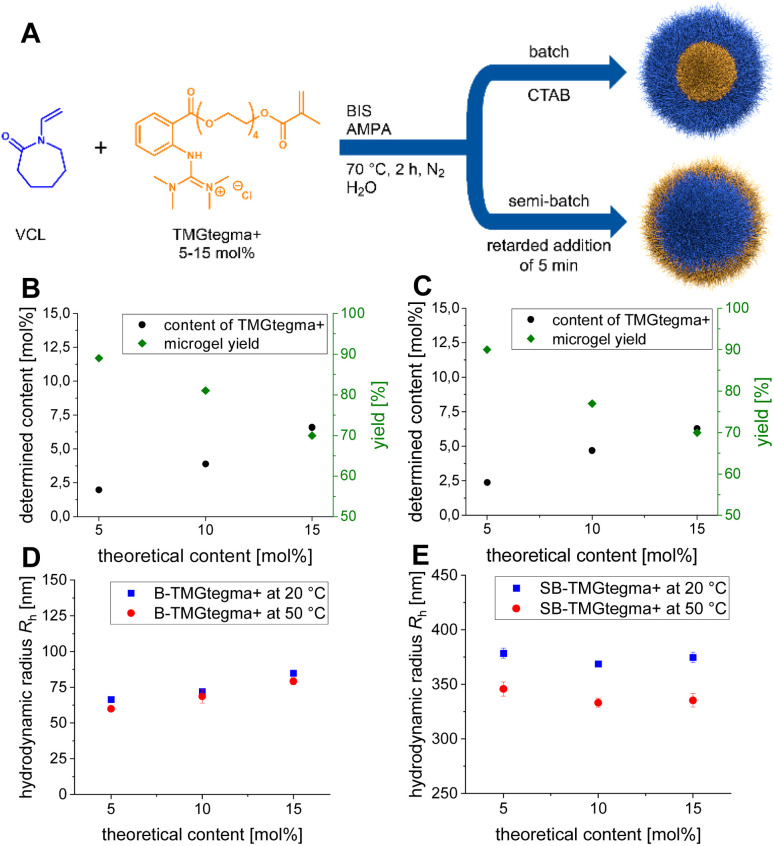
(A) Schematic synthesis of guanidine-modified microgels *via* batch and semi-batch precipitation polymerization. Gravimetrically calculated yields and TMGtegma+ contents of the B-TMGtegma+ (B) and SB-TMGtegma+ (C) microgels determined *via*^1^H NMR spectroscopy. Hydrodynamic radii of the B-TMGtegma+ (D) and SB-TMGtegma+ (E) microgels in methanol at 20 and 50 °C measured *via* DLS.

The synthesized protonated tetramethyl guanidine tetraethylene glycol methacrylate hydrochloride (TMGtegma+) monomer was used as a comonomer to obtain microgels based on poly(*N*-vinylcaprolactam) (PVCL) with 5, 10, and 15 mol% comonomer content. Here, *N*,*N*′*-*methylenebis(acrylamide) (BIS) was used as crosslinker and 2,2′-azobis(2-methylpropionamidine) dihydrochloride (AMPA) as initiator. By batch precipitation polymerization (B-TMGtegma+), the localization of TMGtegma+ in the core of the microgel was achieved, due to the faster reaction of the methacrylate group compared to the vinyl group of VCL,^78–80^ and additionally the surfactant cetyltrimethylammonium bromide (CTAB) was used for the stabilization of growing microgels during the reaction. To enrich TMGtegma+ in the shell of the microgels, semi-batch precipitation polymerization (SB-TMGtegma+) was used and the comonomer was added 5 min after initiation (Fig. 1A). The microgels were then aqueous worked-up so that the protonated form of the guanidine-modified comonomer was retained in the microgels.

Thereafter, the comonomer content in the microgels were determined by nuclear magnetic resonance (NMR) spectroscopy using the internal standard maleic acid. This revealed an evident trend for the batch (Fig. 1B) and semi-batch (Fig. 1C) microgels. The incorporated content increases with increasing theoretical comonomer content, but only about half of the targeted guanidine groups were incorporated independently of the precipitation polymerization mode. For the batch microgels, a lowest content of 2.0 mol% was determined for the B-TMGtegma+ 5 mol% and the highest content of 6.6 mol% for B-TMGtegma+ 15 mol% (Table S2, SI). Similar contents were also found for the semi-batch microgels, ranging between 2.4 and 6.3 mol% (Table S2, SI). Furthermore, the yield of batch and semi-batch microgels decreases with increasing TMGtegma+ content (*cf.*Fig. 1B and C). The yield of batch microgels decreases from 88 to 70% and that of semi-batch microgels from 90 to 70% (Table S1, SI). Therefore, the decrease in both modes corresponds approximately to a decrease of 10% with increasing 5 mol% TMGtegma+ content.

Subsequently, the influence of the different comonomer contents on the hydrodynamic radii (*R*_h_) of the microgels in water at 20 °C was examined *via* dynamic light scattering (DLS). For the batch microgels, the hydrodynamic radii are between 45 and 65 nm with high polydispersity indices (PDIs) (Table S3, SI). The radii increase slightly with increasing TMGtegma+ content. Contrary to this, the microgels containing TMGtegma+ in the shell exhibit significant differences in the hydrodynamic radii at 20 °C, which seem to be influenced by the different TMGtegma+ contents, although no definite trend can be identified. SB-TMGtegma+ 15 mol% has the largest hydrodynamic radius followed by the microgels with 5 mol% TMGtegma+ and the smallest hydrodynamic radii has SB-TMGtegma+ 10 mol% (*cf.* Table S3, SI).

In water, PVCL chains have a lower critical solution temperature (LCST) at 32 °C ^81,82^ and in methanol, the microgels exhibit no volume phase transition temperature (VPTT), in which the following depolymerization studies take place, but will remain in the same order of magnitude even at higher temperatures.^75,83^ To confirm this for the guanidine-modified microgels, the hydrodynamic radii were measured at 20 and 50 °C in methanol. The batch microgels show sizes between 60 and 80 nm independent of the measured temperatures and thus have no VPTT in methanol (Fig. 1D and Table S4, SI). Similar observations are made for the semi-batch microgels. Here, the hydrodynamic radii are around 370 nm at 20 °C and at 50 °C the hydrodynamic radii become slightly smaller at around 340 nm (Fig. 1E and Table S4, SI). Still, this size change is marginal, and it can be concluded that the semi-batch microgels in methanol do not have a VPTT either.

To further investigate the particle size and the influence of the different TMGtegma+ contents on the morphology of the synthesized microgels, bright-field scanning transmission electron microscopy (BFSTEM) images were recorded (Fig. S1, SI). A narrow size distribution is not observed for all comonomer contents of the batch microgels. Thus, a high polydispersity of small spherical particles is observed for the B-TMGtegma+ 5 mol% (Fig. S1A, SI) and microgels with 15 mol% TMGtegma+ show few particles with a lower polydispersity (Fig. S1C, SI). On the other hand, a narrow size distribution with a particle size of 489.2 ± 18.3 nm is found for the B-TMGtegma+ 10 mol% microgels (Fig. S1B and Table S5, SI). Consequently, the amount of surfactant CTAB used seems to be an advantage just for the synthesis of the microgels with 10 mol% TMGtegma+, whereas for the other batch microgel approaches CTAB did not induce any recognizable stabilization. In contrast, spherical particles with a narrow size distribution were observed for the semi-batch microgels regardless of the comonomer content (Fig. S1D–F, SI). With increasing TMGtegma+ content, the microgels become smaller from over 400 nm to less than 280 nm (*cf.* Table S5, SI). Furthermore, an increase in the contrast of the microgels with increasing comonomer content is observed. In addition, the SB-TMGtegma+ 5 mol% exhibit a broad, fuzzy outer layer (Fig. S1D, SI), which becomes smaller as the TMGtegma+ content increases until it is almost undetectable for the SB-TMGtegma+ 15 mol% (Fig. S1F, SI). These findings indicate a lower crosslinking of the shell of the microgels with lower TMGtegma+ contents and a higher crosslinking density in the microgels with increasing content.

### Catalytic performance of microgels in depolymerization of PLA

The synthesized microgels were screened for their catalytic activity in the methanolysis of PLA. Therefore, PLA powder (cryo-milled to 0.75 mm) and the catalyst (guanidine-modified microgel or guanidine monomer) with a loading of 0.5 mol% were placed in a screw cap Schlenk tube and mixed with methanol. The reaction mixture was heated to a chosen temperature for a specific reaction time (see Materials and methods for further information). For quantification, each reaction run was analyzed *via*^1^H NMR spectroscopy and the signal of the methyl lactate methine was compared with the signal of the internal methine of PLA (Fig. S2, SI). Based on this, the conversion of PLA (*X*(int)), the selectivity towards methyl lactate (*S*(MeLa)), and the yield of methyl lactate (*Y*(MeLa)) can be calculated.

In a first step, the optimal reaction temperature was determined under inert conditions using B-TMGtegma+ 15 mol% and SB-TMGtegma+ 15 mol% as catalysts showing at the same time the influence of the guanidine localization within the microgel on the catalytic performance. For the optimization of the temperature, reactions were carried out at temperatures between 50 and 130 °C in steps of 20 °C (Table S6, SI). While at lower temperatures only moderate yields below 15% are achieved, the yield increases rapidly at higher temperatures and reaches a plateau at 110 °C with yields of 94 and 95% for B-TMGtegma+ 15 mol% and SB-TMGtegma+ 15 mol%, respectively (Fig. S4, SI). Thus, the methanolysis of PLA applying guanidine-modified microgels only occurs at elevated temperatures and 110 °C was chosen as the optimal reaction temperature. Here, the localization of the catalyst within the microgel has only a minor influence on the reaction, revealing slightly higher yields with the guanidine located in the core. Also, influences due to diffusion limitations of the microgels are negligible, since PVCL microgels in methanol show no VPTT behavior (*cf.*Fig. 1D and E).^75,83^

With the same microgels, time-dependent measurements were performed to determine the optimized reaction time (Fig. S5 top and Table S7, SI). For B-TMGtegma+ 15 mol%, the yield of methyl lactate follows a saturation curve running into a plateau after 8 hours, while for SB-TMGtegma+ 15 mol% a sigmoidal curve is observable reaching a plateau only after 16 hours. Thus, the reaction rate for microgels containing the guanidine catalyst in the shell is slightly lower than for those containing the guanidine in the core. This correlation is also visible when looking at the time-dependent measurements for all guanidine-modified microgels (Table S7, SI). All microgels obtained from batch precipitation polymerization show high conversions and yields after short reaction times (Fig. S5 middle, SI). Contrary, those obtained from semi-batch precipitation polymerization exhibit a retardation to elongated reaction times (Fig. S5 bottom, SI). In the following, an optimized reaction time of 6 hours was chosen where all catalysts show a full conversion of PLA, but an incomplete methyl lactate production allowing for a more detailed comparison of the guanidine-modified microgels.

At this stage, the product mixture of the reaction with the highest selectivity towards and yield of methyl lactate applying B-TMGtegma+ 10 mol% after 24 hours (Table S7, Entry 8, SI) was further investigated to obtain insides on the methyl lactate separation and guanidine catalyst stability within the microgel. Therefore, all volatile components of the reaction mixture were collected in a cold trap and afterwards methyl lactate was distilled off (see Materials and methods for further information). After distillation, the ^1^H NMR spectrum of methyl lactate still shows an impurity of methanol (Fig. S6 top, SI). This might be due to the challenging purification of the small amount of product mixture in the distillation apparatus. In future, scaling up the methanolysis of PLA catalyzed by the guanidine-modified microgels will allow for the recovery of methyl lactate in high purity and yields, as already shown in literature for other systems.^45,53^ The remaining microgel in the reaction vessel was dried under reduced pressure and, afterwards, analyzed by ^1^H NMR spectroscopy using the internal standard maleic acid. Compared to the spectrum of the B-TMGtegma+ 10 mol% microgel before being used for the depolymerization of PLA (Fig. S6 middle, SI), the spectrum after depolymerization shows oligomer residues and small amounts of methanol and methyl lactate (Fig. S6 bottom, SI). Thus, despite complete reaction (Table S7, Entry 8, SI) and thorough drying, impurities still remain in the microgel preventing a quantitative analysis of the comonomer amount after the depolymerization. However, an approximate comparison of the guanidine content in the microgel is feasible by determining the internal ratio between a guanidine signal and a VCL signal (see blue boxes, Fig. S6 middle and bottom, SI). The comparison shows similar values which indicates no leaching of the guanidine catalyst from the microgel due to the depolymerization. The purification of the microgel after the reaction could be further optimized, *e.g.* by using tangential flow filtration (TFF) equipment as previously reported.^75^

With the optimized reaction conditions (110 °C, 6 hours reaction time), threefold determinations were conducted (Table 1). The results of all single experiments are displayed in the SI (Tables S8 and S9). Under inert conditions, a decrease of the selectivity towards and yield of methyl lactate is observable for the B-TMGtegma+ microgels (Table 1, Entry 1–3) as well as for the SB-TMGtegma+ microgels (Entry 4–6): while applying B-TMGtegma+ 5 mol%, the highest yield for all investigated microgels is reached with 92 ± 4%, which slightly decreases to 90 ± 1% with B-TMGtegma+ 10 mol% and even further to 83 ± 3% with B-TMGtegma+ 15 mol%. Likewise, SB-TMGtegma+ 5 mol% shows the highest yield for all semi-batch microgels with 78 ± 4%, followed by SB-TMGtegma+ 10 mol% with 60 ± 1% and SB-TMGtegma+ 15 mol% with 52 ± 5%. While the B-TMGtegma+ microgels show only a small decrease with increasing guanidine content and are in the same order of magnitude taking the error into account, the differences are more pronounced for the SB-TMGtegma+ microgels. In general, lower yields are obtained when the guanidine catalyst is located in the shell of the microgels compared to those with the guanidine moieties in the core.

**Table 1:** Conversion of PLA (*X*(int)), selectivity towards methyl lactate (*S*(MeLa)), and yield of methyl lactate (*Y*(MeLa)) for the methanolysis of PLA using guanidine-modified microgels as catalyst.^a^ Average values of a threefold determination with standard deviation are displayed

Entry	Catalyst	N_2_/air	*X*(int) [%]	*S*(MeLa) [%]	*Y*(MeLa) [%]
1	B-TMGtegma+ 5 mol%	N_2_	100 ± 1	93 ± 4	92 ± 4
2	B-TMGtegma+ 10 mol%	N_2_	99 ± 1	91 ± 2	90 ± 1
3	B-TMGtegma+ 15 mol%	N_2_	98 ± 1	84 ± 4	83 ± 3
4	SB-TMGtegma+ 5 mol%	N_2_	98 ± 2	79 ± 3	78 ± 4
5	SB-TMGtegma+ 10 mol%	N_2_	97 ± 2	62 ± 1	60 ± 1
6	SB-TMGtegma+ 15 mol%	N_2_	98 ± 1	53 ± 5	52 ± 5
7	TMGtegma+	N_2_	7 ± 1	38 ± 6	2 ± 1
8	B-TMGtegma+ 5 mol%	Air	98 ± 1	75 ± 13	73 ± 13
9	B-TMGtegma+ 15 mol%	Air	98 ± 1	70 ± 9	68 ± 9
10	SB-TMGtegma+ 5 mol%	Air	98 ± 1	64 ± 6	63 ± 5
11	SB-TMGtegma+ 15 mol%	Air	99 ± 1	48 ± 4	47 ± 5
12	TMGtegma+	Air	13 ± 5	38 ± 3	5 ± 2

a Standard procedure: screw cap Schlenk tube, N_2_ or air atmosphere, 110 °C, 6 h, 260 rpm, 0.50 mol% catalyst loading (regarding the polymer ester bond in PLA and corresponding to the guanidine units within the microgel), MeOH (2.00 mL, 14.2 equiv.), PLA (250 mg, 1.00 equiv., bio-mi Ltd).

**Fig. 2:**
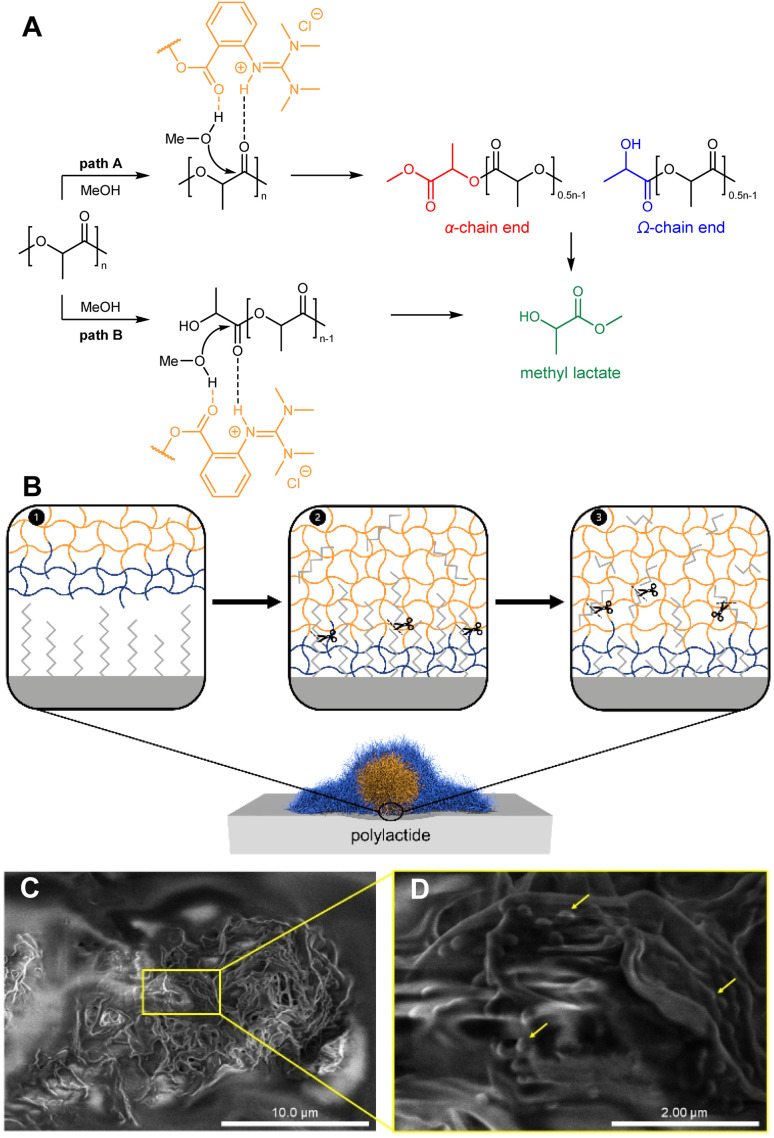
(A) Possible mechanism during depolymerization of PLA *via* random scission of the chains (path A) or *via* cutting of the chain ends (path B).^27,45,59^ (B) Potential three-step depolymerization occurring in the batch microgels: first step (1) penetration of the free PLA chains into microgel, second step (2) breaking of the chains into oligomers, third step (3) oligomers are converted to methyl lactates. (C) Cryo-SEM images of PLA depolymerization using SB-TMGtegma+ 15 mol% microgels after 2 h reaction time with enlarged view (D) of the microgels (arrows) on the PLA surface.

This outcome seems counterintuitive as the catalyst located in the shell should be more accessible, no diffusion limitation should occur and consequently should lead to higher yields. However, the enhanced catalytic activity of the B-TMGtegma+ microgels can be explained with the depolymerization mechanism (Fig. 2).^45,59^ The depolymerization of PLA can either occur *via* random scission of the chains (Fig. 2A, path A) or *via* cutting of the chain ends (path B). Random scission leads to the formation of oligomers, while latter mechanism directly converts PLA to methyl lactate.^27^ As ^1^H NMR spectroscopy reveals the presence of oligomers, guanidine-modified microgel catalyzed depolymerization proceeds *via* random scission of the chains. Based on these findings, we propose the following three-step depolymerization mechanism occurring in the batch microgels (Fig. 2B): first, the chain ends of the free PLA penetrate into the microgel, which adheres to the surface of the PLA due to the deformability of the microgels.^84,85^ The adhesion of microgels on the PLA surface was visualized *via* cryo-SEM measurements (Fig. 2C and D) and a control experiment was performed for pure PLA, showing a smooth PLA surface (Fig. S3, SI).^86–89^ The penetration takes place due to the hydrophobicity of the PLA and the preferred accumulation in hydrophobic surroundings such as the microgel core. Second, the penetrating PLA chain ends are cut into oligomers by random scission (marked by the scissor symbol in Fig. 2B (2)). Afterwards, the oligomers remain in the hydrophobic surrounding of the microgel and are converted to methyl lactate by the guanidine catalyst in a last step. The higher density of catalytically active centers in the microgel core thus leads to the local increase in catalyst concentration at the PLA surface and thus to a more efficient depolymerization. On the contrary in SB-TMGtegma+ microgels, penetrating PLA chains are cut into oligomers as well, but the oligomer enrichment in the microgel core promotes the separation of the oligomers from the guanidine catalysts located in the shell. Furthermore, the generally lower density of catalytically active centers in the fuzzier microgel shell leads to a less efficient depolymerization.

In a control experiment, the protonated guanidine monomer TMGtegma+ was used as catalyst (0.5 mol%) in a threefold determination under inert conditions leading to a low PLA conversion and a methyl lactate yield of only 2 ± 1% (Table 1, Entry 7). Therefore, TMGtegma+ shows only a catalytic activity after incorporation into the microgel due to its preferable hydrophobic surroundings for PLA depolymerization. For example, the activity in the depolymerization increases by 90% when comparing TMGtegma+ to B-TMGtegma+ 5 mol%. This highlights the microgel's capability as a catalyst carrier system.

Furthermore, for industrial application, the stability and activity of catalysts under aerobic conditions are of high importance. Therefore, the threefold determination for the batch and semi-batch microgels with the highest and lowest catalyst loadings as well as for the protonated guanidine monomer TMGtegma+ were repeated under aerobic conditions (Table 1, Entry 8–12). While PLA is still fully converted after the reaction time of 6 hours, the yield of methyl lactate applying B-TMGtegma+ microgels decreases up to 19% (Entry 8 compared to Entry 1), whereas the difference accounts up to 15% for the SB-TMGtegma+ microgels (Entry 10 compared to Entry 4). Thus, only a moderate decrease in catalytic activity is observable, which is probably due to the presence of oxygen and humidity in the system. In case of the protonated guanidine monomer TMGtegma+, no significant changes occur. Yet, the error on the threefold determinations is significantly higher than for the depolymerizations under inert conditions revealing an influence of the chosen conditions on the reproducibility of the experiments. However, the results show a reasonable catalytic activity of the guanidine-modified microgels in the depolymerization of PLA even without the need for inert conditions.

### Recycling of catalytic microgels in depolymerization process

After evaluating the catalytic performance of the microgels, their recyclability in the methanolysis of PLA was screened in a recycling study to further investigate their capability as catalyst carrier systems. Therefore, the depolymerization was performed applying the optimized conditions (110 °C, 6 hours reaction time) under air atmosphere. After each reaction run, the volatile components were removed under reduced pressure and to the remaining microgel catalyst were added PLA powder and methanol as reactant to start a new reaction cycle (see Materials and methods for further information).

**Fig. 3:**
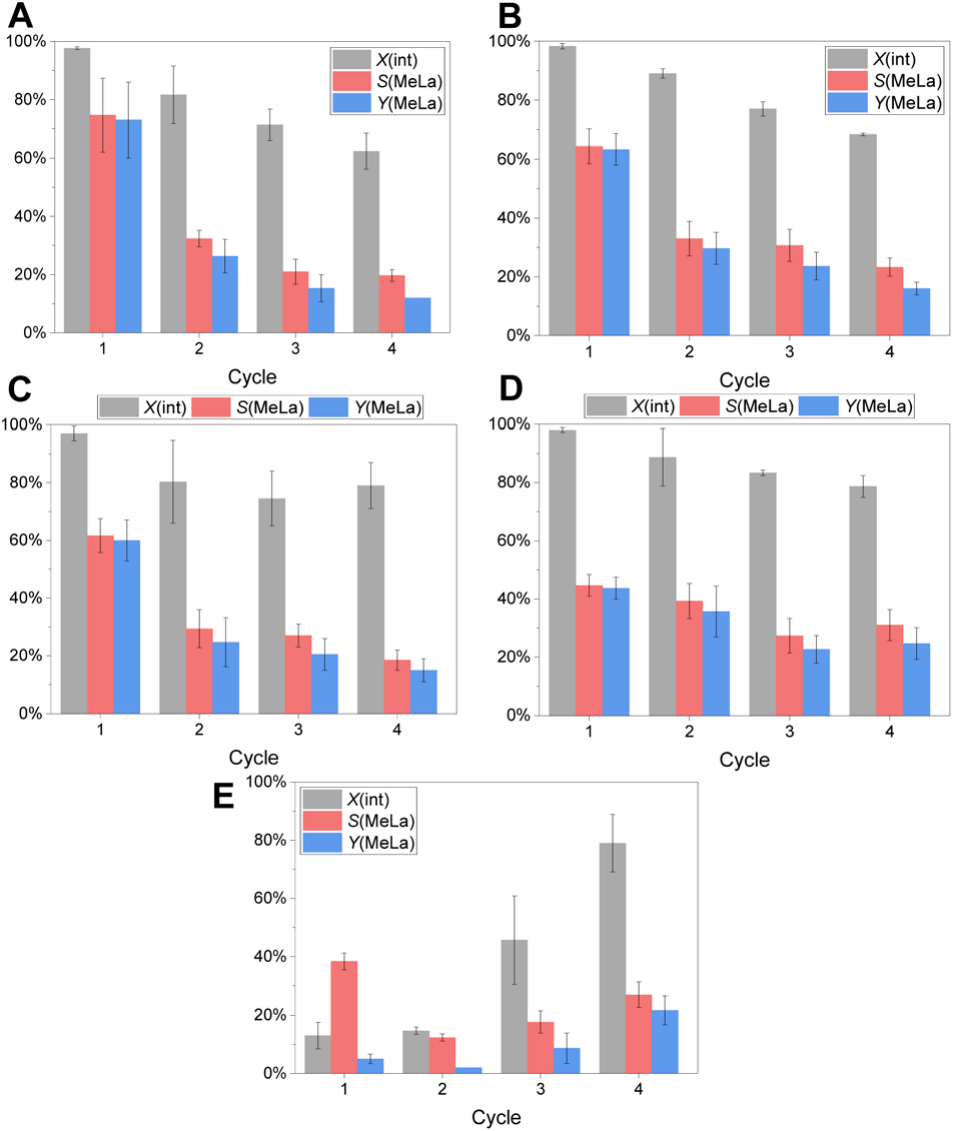
Conversion of PLA (*X*(int), black), selectivity towards methyl lactate (*S*(MeLa), red), and yield of methyl lactate (*Y*(MeLa), blue) for the repeatedly performed methanolysis of PLA under aerobic conditions recycling the catalyst after each reaction cycle. Used catalysts: B-TMGtegma+ 5 mol% (A), SB-TMGtegma+ 5 mol% (B), B-TMGtegma+ 15 mol% (C), SB-TMGtegma+ 15 mol% (D), and as reference experiment TMGtegma+ (E). Average values of a threefold determination with standard deviation are displayed.

The quantification was again performed *via*^1^H NMR analysis (Fig. S2, SI). The recycling of the guanidine-modified microgels was performed in threefold determinations for four reaction cycles each (Fig. 3). The results of all single experiments are displayed in the SI (Tables S10–S14). As discussed in the previous chapter, all applied catalysts lead to a full conversion of PLA in a first reaction cycle, while B-TMGtegma+ 5 mol% (Fig. 3A) and SB-TMGtegma+ 5 mol% (Fig. 3B) show a higher selectivity and yield of methyl lactate compared to the respective microgels B-TMGtegma+ 15 mol% (Fig. 3C) and SB-TMGtegma+ 15 mol% (Fig. 3D) with a higher guanidine content. The reference experiment applying TMGtegma+ (Fig. 3E) leads to the lowest methyl lactate selectivity and yield in the first reaction cycle as discussed above already.

In the consecutive depolymerization reactions (cycles 2–4), all guanidine-modified microgels show a similar behavior. The conversion of PLA decreases stepwise until the fourth reaction cycle and is most pronounced for the microgels with the lowest guanidine content: the decrease accounts to 35% for B-TMGtegma+ 5 mol% (Fig. 3A) and to 30% for SB-TMGtegma+ 5 mol% (Fig. 3B). Likewise, the methyl lactate yield decreases. Exemplarily, the yield drops from 44 ± 4% in the first to 25 ± 5% in the fourth cycle for SB-TMGtegma+ 15 mol% (Fig. 3D), which corresponds to an activity decrease of 43% in total. Again, the most distinct difference is observable for the microgels with the lowest catalyst loading, where the yield decreases from 63 ± 5% to 16 ± 2% (decrease of 75% in total) for SB-TMGtegma+ 5 mol% (Fig. 3B) and even more from 73 ± 13% to 12 ± 0% (decrease of 84% in total) for B-TMGtegma+ 5 mol% (Fig. 3A). In case of the reference experiment applying TMGtegma+, another phenomenon occurs (Fig. 3E). Here, the PLA conversion and methyl lactate yield increase with increasing number of reaction cycles. This is due to the fact that unreacted PLA and oligomers cannot be removed with the volatile compounds after each reaction cycle and therefore remain in the reaction mixture (*cf.* Fig. S6 bottom, SI). Consequently, unreacted PLA chains accumulate over the reaction cycles leading to much higher PLA concentrations than intended which falsifies the results. Likewise, a similar but less pronounced influence will occur and has to be considered for the recycling experiments applying the guanidine-modified microgels from the second reaction cycle on, where no full PLA conversions are reached anymore.

Overall, the guanidine catalyst TMGtegma+ is activated when introducing it into the microgel as a catalyst carrier system as observed for the first depolymerization reaction in the recycling experiments. This is in accordance with our previous reaction condition optimization experiments. In the consecutive reaction cycles, the incorporation of the guanidine moiety into microgels shows a clear benefit as reliable results with a reasonable decrease in catalytic activity are obtained. This highlights the capability of microgels as catalyst carrier systems. In future studies, this capability can be further investigated by scaling up the reaction and conducting it *e.g.* in a continuous flow reactor as we have shown previously for a similar microgel system and a model reaction in a tangential flow filtration (TFF) equipment as a proof of concept.^75^ With this, the guanidine-modified microgels can be further developed to a scalable and recyclable catalyst system to improve circular economy.

## Conclusions

In this study, we successfully synthesized the protonated guanidine-modified monomer tetramethyl guanidine tetraethylene glycol methacrylate (TMGtegma+) as a catalyst for the depolymerization of polylactide (PLA) and incorporated it into microgels as catalyst carrier systems. By utilizing batch or semi-batch precipitation polymerization with varying amounts of TMGtegma+, microgels with different guanidine contents in the core (B-TMGtegma+) or in the shell (SB-TMGtegma+) of the microgels were obtained. The microgels were fully analyzed by DLS and BFSTEM measurements revealing the formation of spherical particles. The guanidine content within the microgels was quantitatively determined *via*^1^H NMR spectroscopy showing a reasonable catalyst incorporation. The microgels with the highest guanidine loading were used to screen optimized reaction conditions for the methanolysis of PLA, before performing threefold determinations applying all obtained microgel systems. Based on these findings and cryo-SEM measurements of the depolymerization mixture, we proposed a three-step depolymerization mechanism taking place within the microgels. Compared to unsupported TMGtegma+, having yields of ≤5%, the immobilization of the catalyst in microgels leads to yields of ≥47%. This corresponds to a 9 to 46-fold increase in the catalytic activity. Furthermore, the tested guanidine-modified microgels exhibited a high stability and activity under aerobic conditions and therefore do not depend on inert conditions. In the recycling study, the catalytic systems showed only a moderate and thus reasonable deactivation of the catalyst over several cycles highlighting the potential of microgels as catalyst carrier systems. Consequently, the investigated systems represent capable catalysts for the depolymerization of PLA and can be developed further for their utilization in a circular plastics economy.

## Materials and Methods

### Materials

Acetonitrile (MeCN, Sigma Aldrich, 99.8%), 2,2′-azobis(2-methylpropionamidine) dihydrochloride (AMPA, Sigma Aldrich, 97%), *N*,*N*′-methylenebis(acrylamide) (BIS, Sigma Aldrich, 99%), butylated hydroxytoluene (BHT, Thermo Scientific, 99.8%), cetyltrimethylammonium bromide (CTAB, Carl Roth, > 99%), deuterated chloroform (CDCl_3_, Deutero GmbH, 99.80%), deuterium oxide (D_2_O, Deutero GmbH, 99.95%), dichloromethane (DCM, Sigma Aldrich, > 99.8%), deuterated dimethyl sulfoxide (DMSO-d_6_, Deutero GmbH, 99.80%), isatoic anhydride (ISA, Thermo Scientific, 99%), maleic acid (Sigma Aldrich, HPLC grade), methacryloyl chloride (MAC, Sigma Aldrich, 97%), methanol (MeOH, Acros Organics, 99.8%, extra dry over molecular sieve), sodium hydroxide (NaOH, Merck Millipore), tetraethylene glycol (TEG, Sigma Aldrich, 99%), tetrahydrofuran (THF, Sigma Aldrich, ≥ 99.9%), tetramethylurea (Acros Organics, 99%), toluene (Fisher Chemicals, ≥ 99.8%), triethylamine (TEA, Sigma Aldrich ≥ 99.5%), and water (VWR Chemicals, HPLC grade) were used as received. *N*-Vinylcaprolactam (VCL, Sigma Aldrich, 98%) was distilled and recrystallized from hexane before use. PLA powder was provided by bio-mi Ltd (Croatia) and cryo-milled to 0.75 mm by Leibniz HKI (Leibniz Institute for Natural Product Research and Infection Biology, Hans Knöll Institute).

### Synthesis of guanidine-modified monomer

The guanidine-modified monomer was synthesized starting from the water-soluble substance tetraethylene glycol (TEG). First, the covalent bonding was established by one-fold esterification of TEG with methacryloyl chloride (MAC) (Scheme S1, SI). In the next step, the aromatic group was introduced by reaction with isatoic anhydride (ISA) (Scheme S1, SI). The resulting primary amine group was then alkylated with the Vilsmeier salt chloro-*N*,*N*,*N*′,*N*′-tetramethylformamidinium chloride (TMG-VS)^90,91^ to obtain the *N*-((dimethylamino) ((2-(16-methyl-15-oxo-2,5,8,11,14-pentaoxaheptadec-16-enoyl)phenyl)amino)methylene)-*N*-methylmethanaminium chloride (protonated tetramethyl guanidine tetraethylene glycol methacrylate hydrochloride, TMGtegma+) (Scheme S1, SI).

### Synthesis of PVCL-based microgels

All microgels are based on poly(*N*-vinylcaprolactam) and were synthesized *via* precipitation polymerization according to literature.^92^ We used various amounts (5, 10, and 15 mol%) of the guanidine-modified monomer as comonomer, *N*,*N*′-methylenebis(acrylamide) (BIS) as crosslinker, and 2,2′-azobis(2-methylpropionamidine) dihydrochloride (AMPA) as initiator. Comonomer rich-core microgels were synthesized using batch polymerization and semi-batch polymerization was used to localize the comonomer at the periphery of the microgels (see SI).

### Depolymerization of PLA

The used microgel catalyst (17.4 μmol, 0.5 mol% regarding the polymer ester bond in PLA and corresponding to the guanidine units within the microgel) and the PLA powder (250 mg, 3.47 mmol, 1.00 equiv.) were placed in a 10 mL screw cap Schlenk tube. In case of catalytic reactions under an inert nitrogen atmosphere, the Schlenk tube was evacuated three times for five minutes each and flooded with nitrogen at this stage. Afterwards, MeOH (2.00 mL, 49.4 mmol, 14.2 equiv.) was added and the reaction was started by placing the tube into a preheated metal block on a stirrer. The reaction was stirred at 260 rpm at the chosen temperature for a specific reaction time. To stop the reaction, the tube was removed from the metal block and quickly cooled to room temperature with running tap water. For quantification of each reaction run, ^1^H NMR spectra were recorded. Therefore, three drops of the reaction mixture were solved in deuterated chloroform (0.6 mL).

The product mixture of the reaction applying B-TMGtegma+ 10 mol% after 24 hours (Table S7, Entry 8, SI) was further investigated. Here, the volatile components (MeOH as solvent/reactant and methyl lactate as product) were collected in a cold trap applying reduced pressure. Afterwards, methyl lactate was distilled of using a short path distillation apparatus (90 °C, 1 × 10^−2^ mbar). The remaining microgel in the reaction vessel was further dried under reduced pressure. For the determination of the comonomer content after the reaction, a ^1^H NMR spectrum of the microgel in D_2_O using maleic acid as an internal standard was recorded.

For the recovery of the guanidine-modified microgels under aerobic conditions, the volatile components were collected in a cold trap after each reaction cycle. Therefore, reduced pressure was applied on the screw cap Schlenk tubes for several hours without any further temperature control leaving only the microgel in the reaction vessel. To this, PLA (250 mg, 3.47 mmol, 1.00 equiv.) and MeOH (2.00 mL, 49.4 mmol, 14.2 equiv.) were added and the reaction was started anew by placing the Schlenk tube into a preheated metal block applying the optimized reaction conditions (110 °C, 6 h, 260 rpm). The reaction was stopped and an aliquot for ^1^H NMR spectroscopy was taken as described above.

### Nuclear magnetic resonance (NMR) spectroscopy

^1^H and ^13^C NMR spectra were recorded on a Bruker AV400 Spectrometer (Bruker Corporation, Billerica, MA, USA) at 400 MHz and 100 MHz, respectively. These are indicated as follows: chemical shift *δ* (ppm) (multiplicity, number of protons, assignment, constituent). Chemical shifts are reported to the nearest 0.01 ppm for the ^1^H NMR spectra and the nearest 0.1 ppm for the ^13^C NMR spectra. Deuterated chloroform (CDCl_3_, *δ*_H_ = 7.26 ppm, *δ*_C_ = 77.2 ppm), deuterated dimethyl sulfoxide (DMSO-d_6_, *δ*_H_ = 2.50 ppm, *δ*_C_ = 39.5 ppm), or deuterium oxide (D_2_O, *δ*_H_ = 4.79 ppm) were used as solvent for measurements. The comonomer content of the microgels was determined using maleic acid as an internal standard. Quantifications of the methanolysis of PLA were performed using ^1^H NMR spectroscopy with deuterated chloroform as solvent. These ^1^H NMR spectra were recorded on a Bruker Avance III HD 400 or a Bruker Avance Neo 400 nuclear resonance spectrometer at 400 MHz and 25 °C.

### Dynamic light scattering (DLS)

DLS measurements were performed on an ALV/CGS-3 Compact Goniometer System (ALV-Laser Vertriebsgesellschaft mbH, Hessen, Germany) with an ALV/LSE 5004 Tau Digital Correlator. The JDS Uniphase laser was used that operates at *λ* = 632.8 nm. The samples were measured at a fixed scattering angle *θ* = 90°. The intensity time correlation functions were analyzed using cumulant algorithm. All samples were filtered (1.2 μm PET filter, Chromafil®) before the measurements and diluted with HPLC grade water.

### Electron microscopy

Bright-field scanning transmission electron microscopy (BFSTEM) images were captured on an SU9000 ultrahigh resolution SEM (Hitachi High-Technologies, Tokyo, Japan). For BFSTEM images, the microgel samples were diluted to a concentration of 1 mg mL^−1^ and a single droplet was placed onto each carbon coated copper grid (300 Mesh Cu) from Agar Scientific Ltd. The microgel size of the recorded images were determined with the software ImageJ 1.52 t. The cryogenic scanning electron (cryo-SEM) microscopy images were taken on a cold field emission S-4800 FE-SEM (Hitachi High-Technologies, Tokyo, Japan) equipped with a Gatan cryo-Chamber. For analysis, the sample was frozen with liquid nitrogen, placed in a sample holder, and transferred into a preparation chamber under vacuum. The upper layer of the sample was broken off by razor blade, while under cooling with liquid nitrogen inside the preparation chamber. The sample was measured at 2 μA with 1.0 kV with an SE detector.

### Mass spectrometry

Mass spectra were recorded by electrospray ionization (ESI) with high resolution mass spectrometry (HRMS) on a Bruker micrOTOF Q II (Bruker Daltonics, Bremen, Germany) with a source voltage of 4.5 kV. Detection was in positive ion mode and acetonitrile was used as solvent.

## Author contributions

F. F. and F. G. were contributing equally to this work by developing the concept and carrying out experiments. S. O. and P. N. supported synthesis and catalysis reactions. F. F. and F. G. wrote the manuscript with the support of S. O., P. N., A. P., and S. H.-P. All authors reviewed the manuscript.

## Conflicts of interest

There are no conflicts to declare.

## Supplementary Material

SC-OLF-D5SC03443D-s001

## Data Availability

Additionally, original and processed data of the DLS measurements for the microgels and NMR measurements for the methanolysis of PLA are available as a data publication *via* the RADAR4Chem repository by FIZ Karlsruhe – Leibniz Institute for Information Infrastructure and are published under an Open Access model (CC BY-NC 4.0, Attribution-NonCommercial). https://dx.doi.org/10.22000/rmsbn8enm4e4bpf3. The data that support the findings of this study are available in the supplementary material (SI) of this article. Supplementary information is available. See DOI: https://doi.org/10.1039/d5sc03443d.
