# Supplementary material for: Depolymerization of PLA catalyzed by guanidine-modified microgels
Source: Chem Sci. 2025 Sep 23;16(42):19614–23. doi: 10.1039/d5sc03443d (PMC12495307; doi:10.1039/d5sc03443d)
Supplement: SC-016-D5SC03443D-s001 [file SC-016-D5SC03443D-s001.pdf]

Supplementary Information (SI) for Chemical Science.
This journal is © The Royal Society of Chemistry 2025

Supplementary Information

Depolymerization of PLA Catalyzed by Guanidine-Modified Microgels

Fabian Fink,‡[a] Frédéric Grabowski,‡[bc] Sandra Oden,[a] Paul Nisgutski,[bc] Andrij Pich,*[bcd] and Sonja Herres-Pawlis*[a]

‡ These authors contributed equally to the work.

* Corresponding authors: sonja.herres-pawlis@ac.rwth-aachen.de, pich@dwi.rwth-aachen.de

[a] Institute of Inorganic Chemistry, RWTH Aachen University, Aachen, D-52074, Germany

[b] Institute of Technical and Macromolecular Chemistry, RWTH Aachen University, Aachen, D-52074, Germany

[c] DWI – Leibniz Institute for Interactive Materials, Aachen, D-52074, Germany

[d] Aachen Maastricht Institute for Biobased Materials, Maastricht University, RD Geleen, 6167, The Netherlands

Table of Contents

Synthesis of Guanidine-Modified Monomer 2

Synthesis of Guanidine-Modified Microgels 5

Evaluation of PLA Methanolysis 10

Depolymerization of PLA 11

Determination of the Optimized Reaction Temperature 12
Time-Dependent Determinations 13
Threefold Determinations 16
Recycling of the Guanidine-Modified Microgels under Aerobic Conditions 18

References 23

Synthesis of Guanidine-Modified Monomer

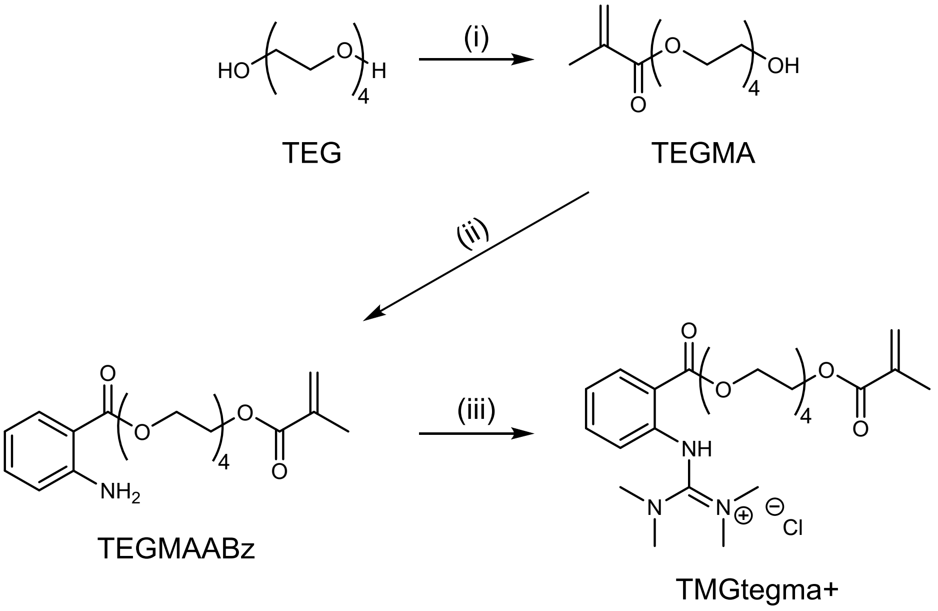

Scheme 1. Synthesis of guanidine-modified monomer. Reagents and conditions: (i) MAC, TEA, BHT, DCM, 0 °C to rt, 24 h; (ii) ISA, NaOH, BHT, THF, 75 °C, 24 h, N_2; (iii) TMG-VS, TEA, BHT, MeCN, 100 °C, 4 h, N_2.

Synthesis of 2-(2-(2-(2-hydroxyethoxy)ethoxy)ethoxy)ethyl methacrylate (tetraethylene glycol methacrylate, TEGMA): TEG (74.5 g, 382.5 mmol), TEA (10.6 mL, 76.5 mmol), and BHT (8.1 mg, 0.1 wt%) were dissolved in DCM (120 mL) and cooled to 0 °C. MAC (8.10 g, 76.5 mmol) was dissolved in DCM (60 mL) and added dropwise to the reaction mixture. After 1 h, the mixture was warmed to r.t. and stirred for 24 h. Thereafter, the mixture was washed with deionized H_2O (3x 180 mL) and organic phase was collected. The solvent of the organic phase was removed *via* rotary evaporation and the crude product was further purified *via* column chromatography on neutral silica gel (ethyl acetate:methanol; 19:1, R_f = 0.48)

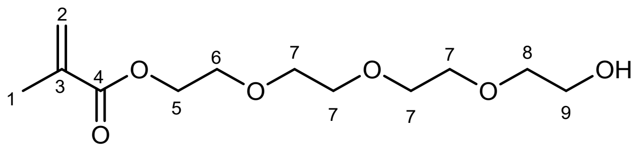

resulting in a yellow oil (12.9 g, 64 %).

^{1}**H NMR** (400 MHz, $CDCl_3$): δ (ppm) = 6.13 (s, 1H, 2), 5.57 (s, 1H, 2), 4.30 (t, 2H, 5), 3.69-3.77 (m, 4H, 6+9), 3.66 (s, 8H, 7), 3.58-3.63 (m, 2H, 8), 1.94 (s, 3H, 1). ^{13}**C NMR** (100 MHz, $CDCl_3$): δ (ppm) = 167.5 (1C, 4), 136.3 (1C, 3), 125.9 (1C, 2), 72.6 (1C, 8), 70.5-70.8 (4C, 7), 69.3 (1C, 6), 64.0 (1C, 5), 61.9 (1C, 9), 18.5 (1C, 1). **HRMS (ESI)** m/z: $[M + H]^+$ Calcd for $C_{12}H_{23}O_6$ 263.1500; Found 263.1489.

Synthesis of 14-methyl-13-oxo-3,6,9,12-tetraoxapentadec-14-en-1-yl 2-aminobenzoate (tetraethylene glycol methacrylate 2-aminobenzoate, TEGMAABz): TEGMA (8.00 g, 30.5 mmol), isatoic anhydride (ISA, 2.99 g, 18.3 mmol), NaOH (0.13 g, 3.1 mmol), and BHT (8.6 mg, 0.1 wt%) were dissolved in THF (160 mL) and degassed by three freeze-pump-thaw cycles. Then, the reaction was performed at 75 °C for 24 h under inert atmosphere. Afterwards, the solvent was removed *via* rotary evaporation and the crude product was further purified *via* column chromatography on neutral silica gel (ethyl acetate:cyclohexane;

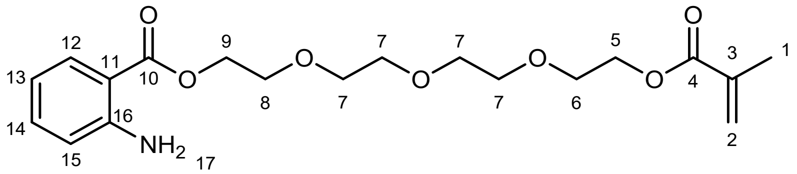

7:3, R_f = 0.85) resulting in a yellow oil (6.3 g, 90 %).

^{1}**H NMR** (400 MHz, DMSO-d6): δ (ppm) = 7.70 (dd, *J* = 1.7 Hz, 1H, 12), 7.24 (t, *J* = 1.7 Hz, 1H, 14), 6.76 (d, *J* = 1.2 Hz, 1H, 15), 6.62 (s, 2H, 17), 6.52 (t, *J* = 1.2 Hz, 1H, 13), 6.02 (s, 1H, 2), 5.67 (s, 1H, 2), 4.31 (t, 2H, 9), 4.19 (t, 2H, 5), 3.72 (t, 2H, 8), 3.63 (t, 2H, 6), 3.55 – 3.49 (m, 8H, 7), 1.87 (s, 3H, 1). ^{13}**C NMR** (100 MHz, DMSO-d6): δ (ppm) = 167.3 (1C, 10), 166.5 (1C, 4), 151.4 (1C, 16), 135.8 (1C, 3), 134.1 (1C, 14), 130.7 (1C, 12), 125.8 (1C, 2), 116.5 (1C, 15), 114.7 (1C, 13), 108.8 (1C, 11), 69.8-69.9 (4C, 7), 68.4 (1C, 6), 68.2 (1C, 8), 63.7 (1C, 9), 63.2 (1C, 5), 17.9 (1C, 1). **HRMS (ESI)** m/z: $[M + H]^+$ Calcd for $C_{19}H_{28}NO_7$ 382.1870; Found 382.1860.

Synthesis of chloro-*N*,*N*,*N′*,*N′*-tetramethylformamidinium chloride (TMG-VS): The Vilsmeier salt TMG-VS was synthesized as described in the literature.[1,2] To a solution of tetramethylurea (50.0 g, 430 mmol) in toluene (200 mL) in a Schlenk flask, phosgene was passed through at 0 °C for 2 h. Subsequently, the phosgene inlet was closed, and the mixture was allowed to warm to r.t. for 24 h with stirring, while the permanently mounted condenser was cooled to -30 °C. The formed precipitate was filtrated and washed three times with diethyl ether. The

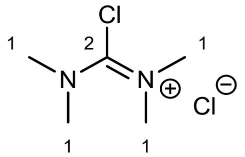

white Vilsmeier salt was dried in vacuo (yield about 95 %).[1,2]

^{1}H NMR (400 MHz, $CDCl_3$): δ (ppm) = 3.49 (s, 12H, 1). **^{13}C NMR** (100 MHz, $CDCl_3$): δ (ppm) = 159.4 (1C, 2), 45.0 (4C, 1).

Synthesis of *N*-((dimethylamino)((2-(16-methyl-15-oxo-2,5,8,11,14-pentaoxaheptadec-16-enoyl)phenyl)amino)methylene)-*N*-methylmethanaminium chloride (protonated tetramethyl guanidine tetraethylene glycol methacrylate hydrochloride, TMGtegma+): Prior to synthesis, all glassware was baked out. First, a solution (1) of TEGMAABz (5.01 g, 13.1 mmol), BHT (5.0 mg, 0.1 wt%), and MeCN (75 mL) was prepared to which then TEA (2.2 mL, 15.7 mmol) was added. A second solution (2) consisting of TMG-VS (2.70 g, 15.7 mmol) and MeCN (75 mL) was prepared and subsequently added to solution (1) under strong stirring. The combined solutions were further degassed by three freeze-pump-thaw cycles. The mixture was then allowed to react for 4 h at 100 °C under inert atmosphere. Afterwards, the solvent was removed *via* rotary evaporation and the crude product was

further purified *via* column chromatography on neutral aluminum oxide (ethyl acetate:methanol; 9:1, R_f = 0.21) resulting in a dark yellow/orange oil (5.0 g, 73 %).

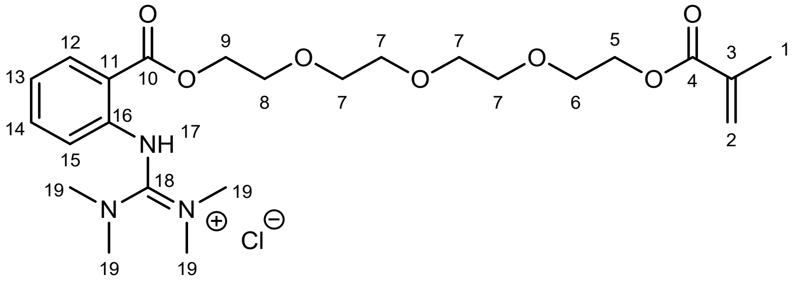

^{1}H NMR (400 MHz, DMSO-d6): δ (ppm) = 10.46 (br s, 1H, 17), 7.71 (d, *J* = 1.6 Hz, 1H, 12), 7.51 (t, *J* = 7.7, 1.6 Hz, 1H, 14), 7.10 (t, *J* = 7.6 Hz, 1H, 13), 7.03 (d, *J* = 8.1 Hz, 1H, 15), 6.02 (s, 1H, 2), 5.68 (s, 1H, 2), 4.29 (t, 2H, 9), 4.19 (t, 2H, 5), 3.70 (t, 2H, 8), 3.63 (t, 2H, 6), 3.54-3.49 (m, 8H, 7), 2.73 (s, 12H, 19), 1.87 (s, 3H, 1). **^{13}C NMR** (100 MHz, DMSO-d6): δ (ppm) = 166.5 (1C, 4), 166.3 (1C, 10), 158.6 (1C, 18), 144.7 (1C, 16), 135.6 (1C, 3), 133.1 (1C, 14), 130.7 (1C, 12), 125.8 (1C, 2), 123.9 (2C, 13+15), 121.8 (1C, 11), 69.8-69.9 (4C, 7), 68.2 (2C, 6+8), 63.7 (2C, 5+9), 39.3 (4C, 19), 18.0 (1C, 1). **HRMS (ESI)** m/z: $[M + Na]^+$ Calcd for $C_{24}H_{37}N_3NaO_7$ 502.2503; Found 502.2524.

Synthesis of Guanidine-Modified Microgels

For comonomer rich core microgels, VCL (amounts see **Table S1**) and BIS (0.285 mmol, 3 mol%) were dissolved in water (131 mL) with CTAB (0.048, 0.5 mol%). TMGtegma+ (amounts see **Table S1**) was first dissolved in water (8 mL) and then added to this solution. Next, the solution was purged with nitrogen for 1 h at 70 °C. Afterwards, a solution consisting of AMPA (0.076 mmol, 0.8 mol%) and water (4 mL) was added to initiate the polymerization. Then, the mixture was stirred (250 rpm) for 2 h at 70 °C. The obtained microgels were dialyzed against deionized water (MWCO: 12-14 kDa) for 5 days, followed by freeze-drying. For the microgels containing comonomer at the periphery, VCL (amounts see **Table S1**) and BIS (0.285 mmol, 3

mol%) were dissolved in water (131 mL). Afterwards, the solution was purged with nitrogen for 1 h at 70 °C under strong stirring. For initiation, AMPA (0.076 mmol, 0.8 mol%) was dissolved in water (4 mL) and added to the reaction mixture. Five minutes after the initiation, a solution consisting of TMGtegma+ (amounts see **Table S1**) in water (8 mL) was added and the mixture was stirred (250 rpm) for 2 h at 70 °C. Likewise, the obtained microgels were dialyzed against deionized water (MWCO: 12-14 kDa) for 5 days and then freeze-dried.

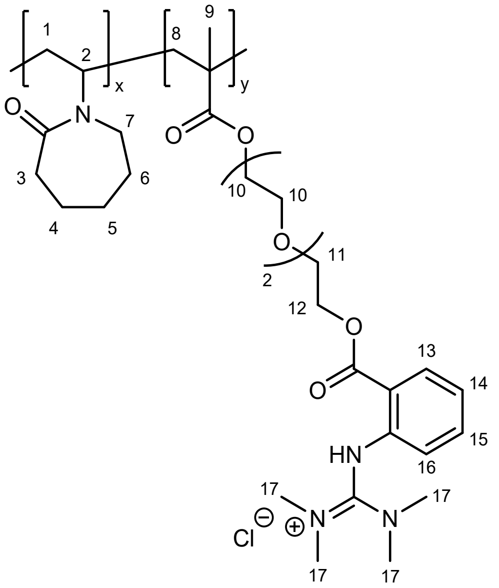

^{1}H NMR (400 MHz, D_2O) of B-TMGtegma+ 5 mol%: δ (ppm) = 8.06 (1H, 13), 7.70 (1H, 15), 7.35 (1H, 14), 7.14 (1H, 16), 4.47 (2H, 12), 3.98-4.90 (1H, 2), 3.89 (2H, 11), 3.68 (12H, 10), 2.84-3.52 (2H, 7), 2.95 (12H, 17), 2.15-2.79 (2H, 3), 0.63-2.12 (13H, 1+4+5+6+8+9).

^{1}H NMR (400 MHz, D_2O) of B-TMGtegma+ 10 mol%: δ (ppm) = 8.06 (1H, 13), 7.69 (1H, 15), 7.35 (1H, 14), 7.14 (1H, 16), 4.47 (2H, 12), 3.97-4.88 (1H, 2), 3.89 (2H, 11), 3.68 (12H, 10), 2.82-3.53 (2H, 7), 2.95 (12H, 17), 2.16-2.77 (2H, 3), 0.74-2.11 (13H, 1+4+5+6+8+9).

^{1}H NMR (400 MHz, D_2O) of B-TMGtegma+ 15 mol%: δ (ppm) = 8.06 (1H, 13), 7.69 (1H, 15), 7.34 (1H, 14), 7.13 (1H, 16), 4.47 (2H, 12), 3.97-4.90 (1H, 2), 3.89 (2H, 11), 3.67 (12H, 10), 2.82-3.53 (2H, 7), 2.93 (12H, 17), 2.16-2.74 (2H, 3), 0.65-2.12 (13H, 1+4+5+6+8+9).

^{1}**H NMR** (400 MHz, D_2O) of SB-TMGtegma+ 5 mol%: δ (ppm) = 8.06 (1H, 13), 7.69 (1H, 15), 7.34 (1H, 14), 7.13 (1H, 16), 4.47 (2H, 12), 3.97-4.87 (1H, 2), 3.89 (2H, 11), 3.68 (12H, 10), 2.84-3.51 (2H, 7), 2.95 (12H, 17), 2.14-2.78 (2H, 3), 0.67-2.10 (13H, 1+4+5+6+8+9).

^{1}**H NMR** (400 MHz, D_2O) of SB-TMGtegma+ 10 mol%: δ (ppm) = 8.04 (1H, 13), 7.68 (1H, 15), 7.33 (1H, 14), 7.12 (1H, 16), 4.46 (2H, 12), 3.95-4.92 (1H, 2), 3.88 (2H, 11), 3.67 (12H, 10), 2.82-3.51 (2H, 7), 2.94 (12H, 17), 2.16-2.78 (2H, 3), 0.69-2.13 (13H, 1+4+5+6+8+9).

^{1}**H NMR** (400 MHz, D_2O) of SB-TMGtegma+ 15 mol%: δ (ppm) = 8.04 (1H, 13), 7.68 (1H, 15), 7.33 (1H, 14), 7.12 (1H, 16), 4.46 (2H, 12), 3.96-4.88 (1H, 2), 3.88 (2H, 11), 3.67 (12H, 10), 2.82-3.47 (2H, 7), 2.94 (12H, 17), 2.16-2.70 (2H, 3), 0.68-2.11 (13H, 1+4+5+6+8+9).

Table S1. Amounts for the TMGtegma+ microgel syntheses and the corresponding gravimetrically determined yields.

**sample**	**m(VCL) [g]**	**m(TMGtegma+) [g]**	**yield [%]**
B-TMGtegma+ 5 mol%	1.258	0.245	88
B-TMGtegma+ 10 mol%	1.190	0.490	81
B-TMGtegma+ 15 mol%	1.125	0.736	70
SB-TMGtegma+ 5 mol%	1.256	0.245	90
SB-TMGtegma+ 10 mol%	1.190	0.490	77
SB-TMGtegma+ 15 mol%	1.125	0.736	70

Table S2. Theoretical TMGtegma+ content and determined TMGtegma+ content of microgels *via* ^{1}H NMR.

**sample**	**TMGtegma+$_{theo.}$ [mol%]**	**TMGtegma+$_{NMR}$ [mol%]**
B-TMGtegma+ 5 mol%	5.0	2.0
B-TMGtegma+ 10 mol%	10.0	3.9
B-TMGtegma+ 15 mol%	15.0	6.6
SB-TMGtegma+ 5 mol%	5.0	2.4
SB-TMGtegma+ 10 mol%	10.0	4.7
SB-TMGtegma+ 15 mol%	15.0	6.3

Table S3. Hydrodynamic radii and PDI of TMGtegma+ microgels in water at 20 °C determined *via* DLS.

sample	R_h 20 °C [nm]	PDI 20 °C
B-TMGtegma+ 5 mol%	49.8 ± 0.6	0.401
B-TMGtegma+ 10 mol%	51.3 ± 0.5	0.435
B-TMGtegma+ 15 mol%	62.7 ± 4.9	0.478
SB-TMGtegma+ 5 mol%	302.6 ± 3.1	0.220
SB-TMGtegma+ 10 mol%	286.5 ± 1.8	0.245
SB-TMGtegma+ 15 mol%	314.2 ± 1.9	0.407

Table S4. Hydrodynamic radii and PDI of TMGtegma+ microgels in methanol at 20 and 50 °C determined *via* DLS.

sample	R_h 20 °C [nm]	PDI 20 °C	R_h 50 °C [nm]	PDI 50 °C
B-TMGtegma+ 5 mol%	66.5 ± 0.1	0.404	59.9 ± 0.7	0.412
B-TMGtegma+ 10 mol%	72.0 ± 0.6	0.454	68.6 ± 4.7	0.479
B-TMGtegma+ 15 mol%	84.9 ± 2.2	0.493	79.2 ± 2.6	0.490
SB-TMGtegma+ 5 mol%	378.6 ± 4.6	0.114	345.8 ± 6.5	0.164
SB-TMGtegma+ 10 mol%	368.9 ± 1.7	0.110	333.2 ± 4.2	0.066
SB-TMGtegma+ 15 mol%	374.8 ± 4.8	0.210	335.4 ± 6.3	0.075

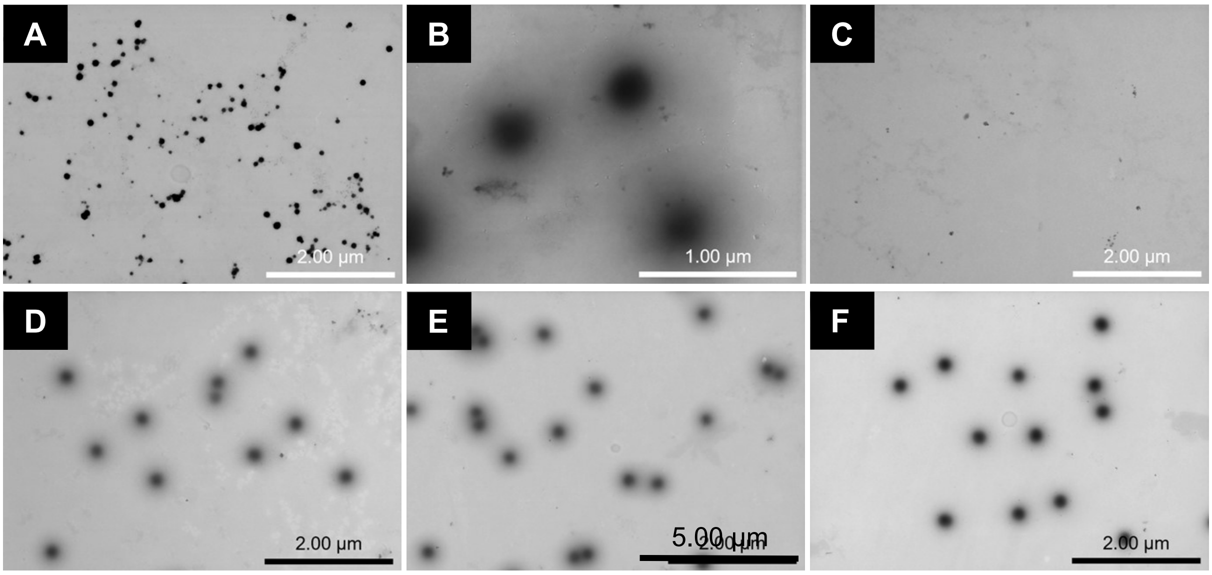

Fig. S1. At the top are the BFSTEM images of the B-TMGtegma+ microgels with 5 mol% (A), 10 mol% (B), and 15 mol% (C). At the bottom are the BFSTEM images of the SB-TMGtegma+ microgels with 5 mol% (D), 10 mol% (E), and 15 mol% (F).

Table S5. Diameter of the B-TMGtegma+ 10 mol% and SB-TMGtegma+ microgels with various comonomer content determined *via* BFSTEM images.

sample	diameter [nm]
B-TMGtegma+ 10 mol%	489.2 ± 18.3
SB-TMGtegma+ 5 mol%	408.9 ± 36.5
SB-TMGtegma+ 10 mol%	334.3 ± 17.8
SB-TMGtegma+ 15 mol%	279.2 ± 16.8

Evaluation of PLA Methanolysis

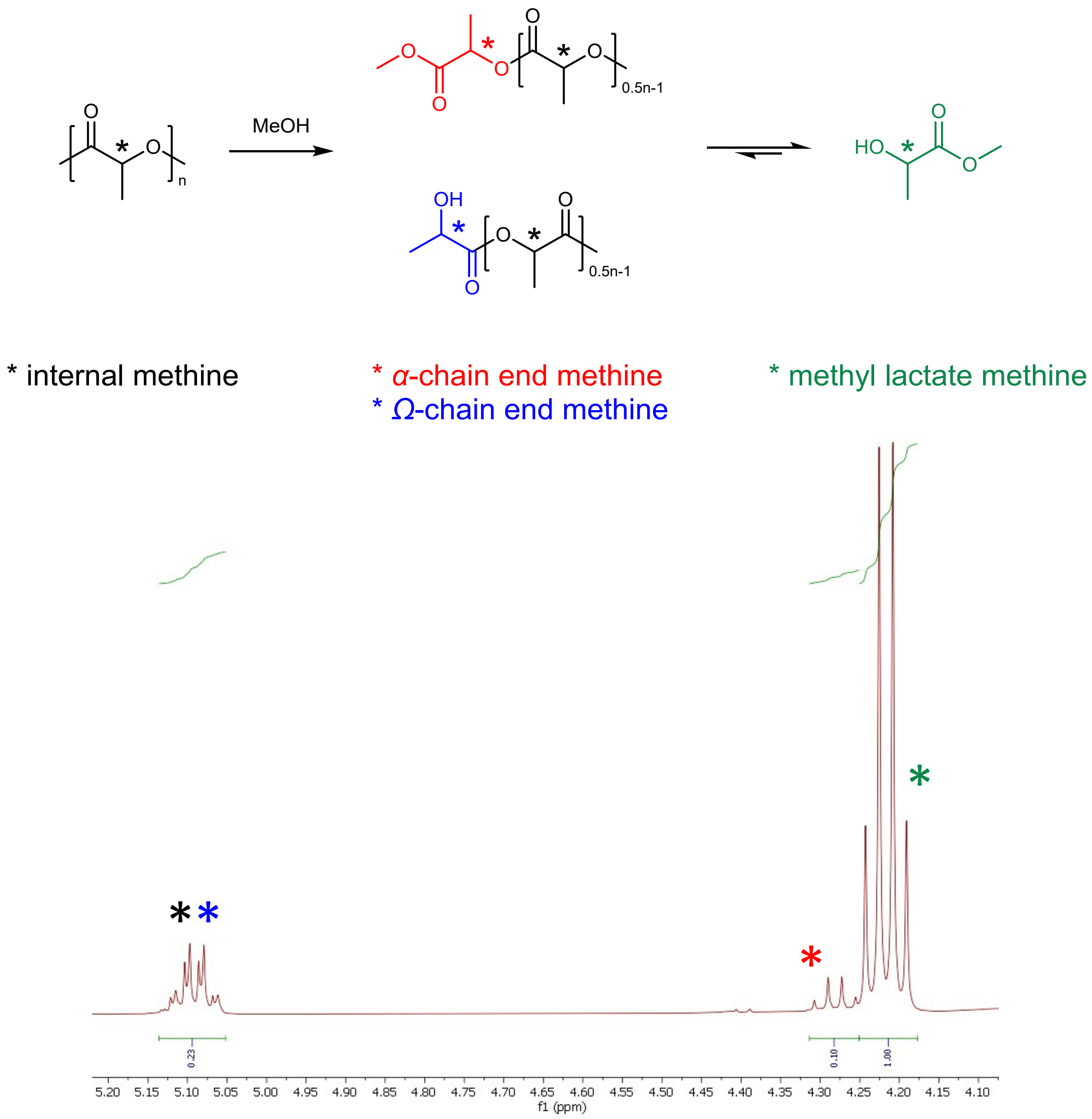

Fig. S2. Schematic PLA methanolysis (top) highlighting the relevant methine moieties for the evaluation of the reaction and an extract of an exemplary ^{1}H NMR spectrum (bottom) with assigned signals; black label: internal methine group of the polymer (5.10 ppm), blue label: methine group of the *Ω*-chain end oligomers (5.10 ppm), red label: methine group of the *α*-chain end oligomers (4.28 ppm), and green label: methine group of the methyl lactate (4.22 ppm).

The data obtained for PLA methanolysis were analyzed according to literature.[3–5] As the signals of the internal methine groups of the polymer (black label) and the methine groups of the *Ω*-chain end oligomers (blue label) overlap in the ^{1}H NMR spectrum, the integral of the internal methine groups has to be calculated following equation 1 as the integrals of the *Ω*- and *α*-chain end methine groups are identical. Consequently, the conversion of PLA (*X*(int), equation 2), the selectivity towards methyl lactate (*S*(MeLa), equation 3), and the yield of methyl lactate (*Y*(MeLa), equation 4) can be calculated.

$$[Int] = [Int/\Omega - end] - [\alpha - end] \quad (1)$$

$$\text{as } [\Omega - end] = [\alpha - end]$$

$$X(int) = 1 - \frac{[Int]}{[Int]_0} \quad (2)$$

$$S(MeLa) = \frac{[MeLa]}{[Int]_0 - [Int]} \quad (3)$$

$$Y(MeLa) = X(int) \cdot S(MeLa) \quad (4)$$

Depolymerization of PLA

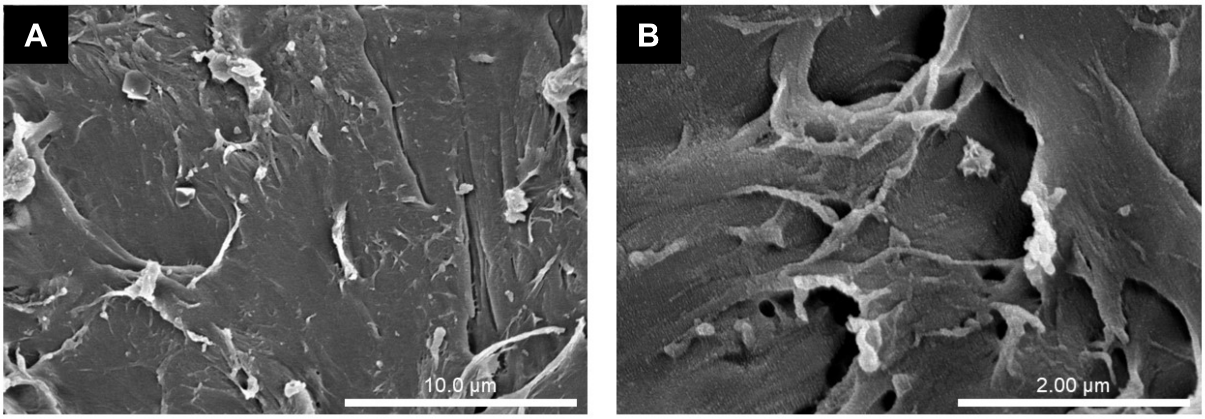

Fig. S3. Cryo-SEM images of the PLA surface as an overview (A) and in enlarged view (B) without microgel after 2 h in methanol at 110 °C. The surface of pure PLA is smooth and exhibits typical fracture morphology with few fibrils.[6–9]

Determination of the Optimized Reaction Temperature

Table S6. Conversion of PLA (*X*(int)), selectivity towards methyl lactate (*S*(MeLa)), and yield of methyl lactate (*Y*(MeLa)) for the methanolysis of PLA using B- or SB-TMGtegma+ 15 mol% as catalyst at varying temperatures determined from onefold measurements.[a]

entry	catalyst	*T* [°C]	*t* [min]	*X*(int) [%]	*S*(MeLa) [%]	*Y*(MeLa) [%]
**1**		50	960	20	31	6
**2**		70	960	30	45	14
**3**	B-TMGtegma+ 15 mol%	90	973	100	93	93
**4**		110	960	100	94	94
**5**		130	960	100	96	96
**6**		50	960	27	8	2
**7**		70	960	24	26	6
**8**	SB-TMGtegma+ 15 mol%	90	969	99	75	74
**9**		110	960	99	96	95
**10**		130	960	100	91	91

[a] Standard procedure: screw cap Schlenk tube, N_2 atmosphere, 260 rpm, 0.50 mol% catalyst loading (regarding the polymer ester bond in PLA and corresponding to the guanidine units within the microgel), MeOH (2.00 mL, 14.2 equiv), PLA (250 mg, 1.00 equiv, bio-mi Ltd.).

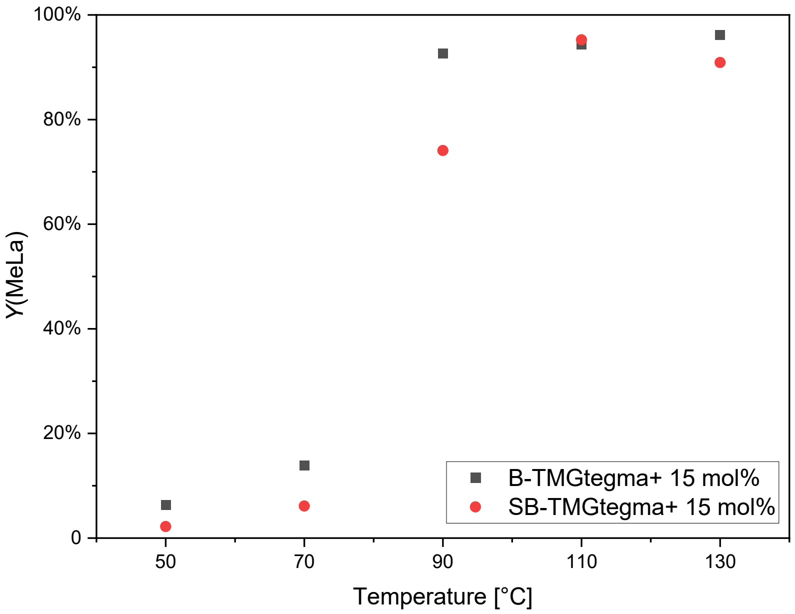

Fig. S4. Graphical representation of the yield of methyl lactate (*Y*(MeLa)) for the methanolysis of PLA using B- or SB-TMGtegma+ 15 mol% as catalyst at varying temperatures after 16 hours.

Time-Dependent Determinations

Table S7. Conversion of PLA (*X*(int)), selectivity towards methyl lactate (*S*(MeLa)), and yield of methyl lactate (*Y*(MeLa)) for the methanolysis of PLA using guanidine-modified microgels as catalyst after varying times determined from onefold measurements.[a]

entry	catalyst	*t* [min]	*X*(int) [%]	*S*(MeLa) [%]	*Y*(MeLa) [%]
**1**	B-TMGtegma+ 5 mol%	120	93	62	57
**2**		240	99	85	84
**3**		360	100	91	91
**4**		1440	100	98	98
**5**	B-TMGtegma+ 10 mol%	120	100	57	57
**6**		240	98	77	75
**7**		360	99	93	92
**8**		1440	100	99	99
**9**	B-TMGtegma+ 15 mol%	120	91	34	31
**10**		240	100	70	70
**11**		360	99	88	87
**12**		480	100	93	93
**13**		960	100	94	94
**14**		1440	100	93	93
**15**	SB-TMGtegma+ 5 mol%	120	54	16	9
**16**		240	95	35	34
**17**		360	98	77	75
**18**		1440	99	83	83
**19**	SB-TMGtegma+ 10 mol%	120	89	31	28
**20**		240	95	36	34
**21**		360	99	60	59
**22**		1440	99	98	97
**23**	SB-TMGtegma+ 15 mol%	120	50	14	7
**24**		240	79	26	20
**25**		360	100	45	45
**26**		480	99	81	80
**27**		960	99	96	95
**28**		1440	99	96	95

[a] Standard procedure: screw cap Schlenk tube, N_2 atmosphere, 110 °C, 260 rpm, 0.50 mol% catalyst loading (regarding the polymer ester bond in PLA and corresponding to the guanidine units within the microgel), MeOH (2.00 mL, 14.2 equiv), PLA (250 mg, 1.00 equiv, bio-mi Ltd.).

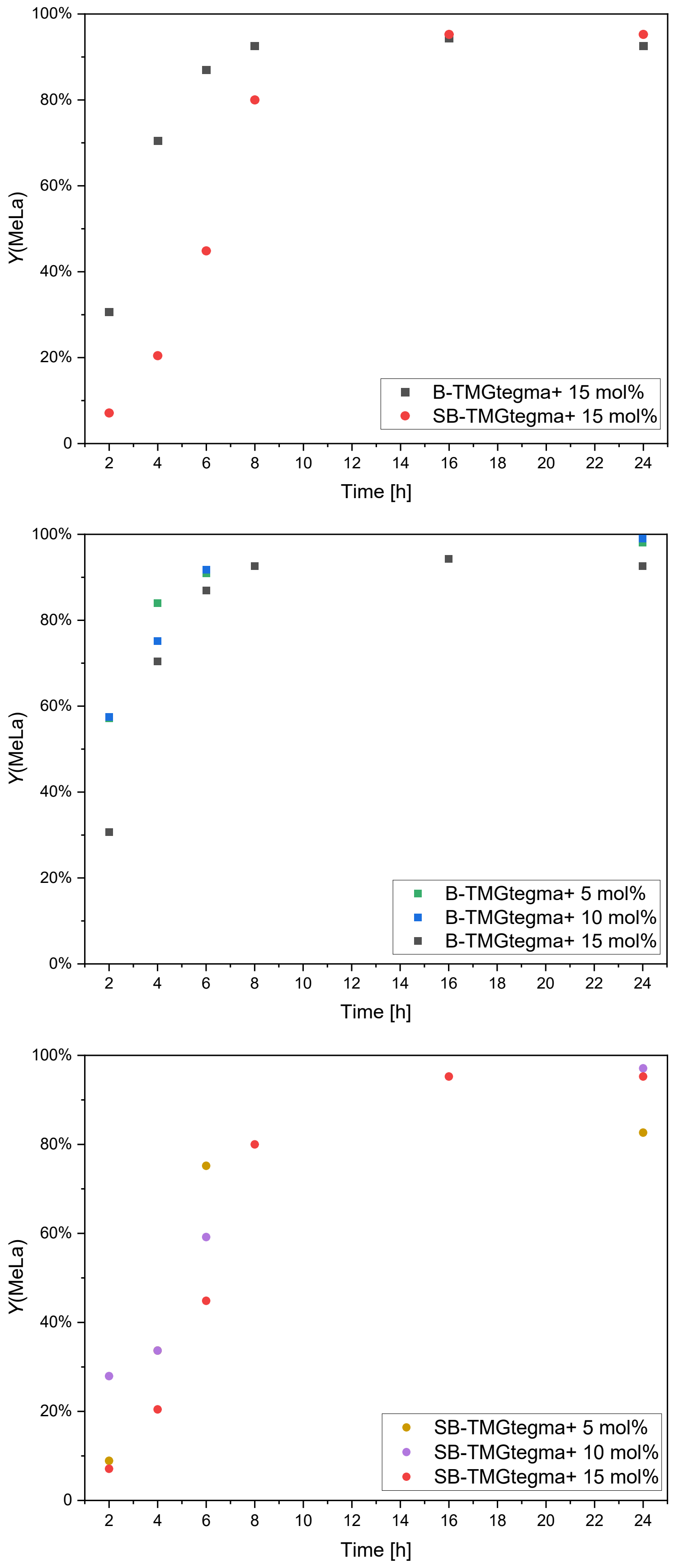

Fig. S5. Graphical representation of the yield of methyl lactate (*Y*(MeLa)) for the methanolysis of PLA using B- or SB-TMGtegma+ 15 mol% as catalyst (top) and comparison of all batch (middle) and semi batch (bottom) microgels at 110 °C after varying times.

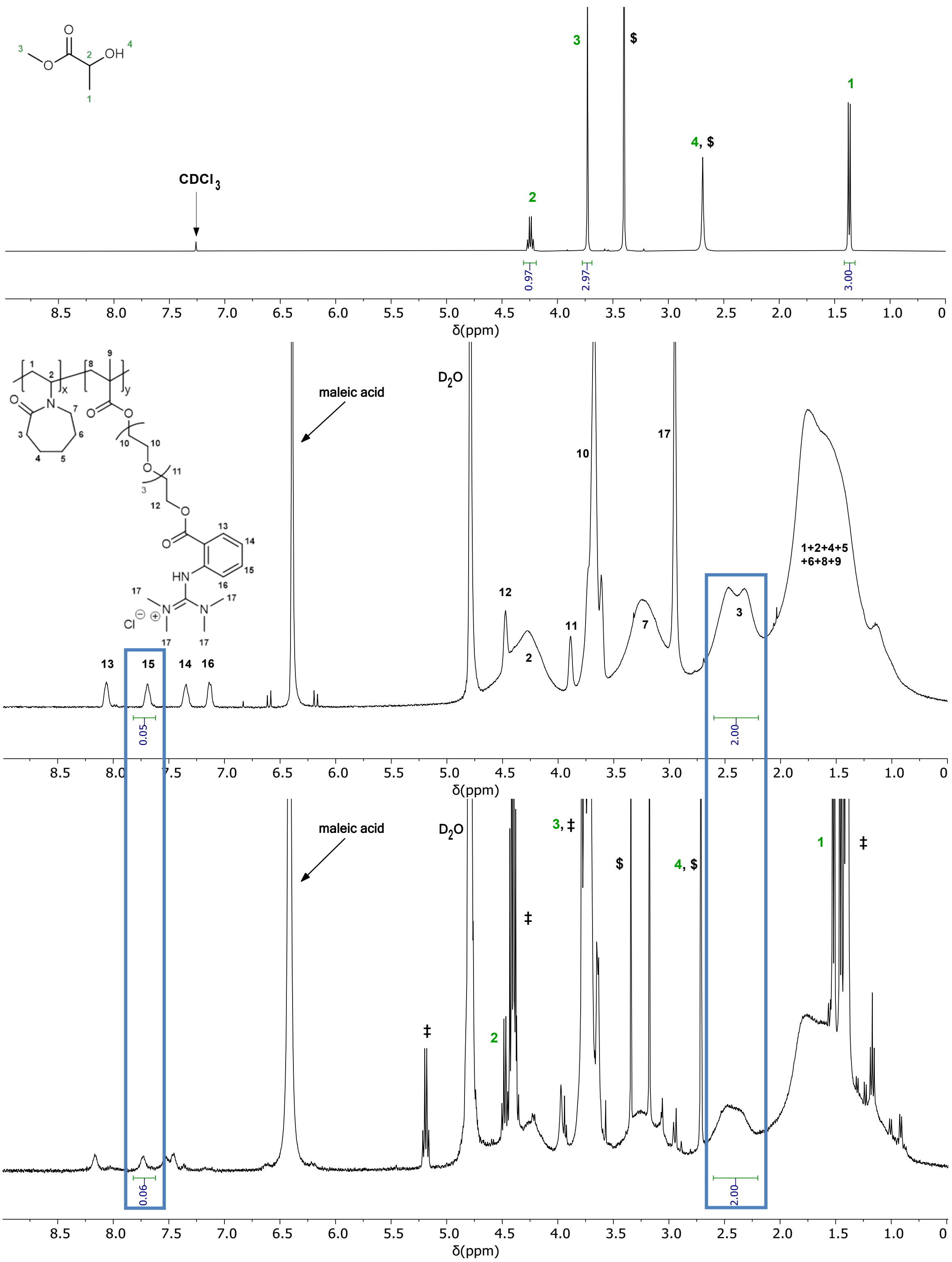

Fig. S6. (Top) ^{1}H NMR spectrum of distilled methyl lactate with minor impurity of methanol ($) after depolymerization of PLA using B-TMGtegma+ 10 mol% microgels as catalyst measured in $CDCl_3$. ^{1}H NMR spectra of B-TMGtegma+ 10 mol% microgel before (middle) and after (bottom) being used for depolymerization of PLA with internal standard maleic acid measured in D_2O. After depolymerization (bottom), oligomer residues (‡) and small amounts of methanol ($) and methyl lactate remain in microgel. For approximate comparison of guanidine content in microgel, integral ratio between a guanidine signal (7.62-7.82 ppm) and a VCL signal (2.20-2.60 ppm) are compared, showing similar values.

Threefold Determinations

Table S8. Conversion of PLA (X(int)), selectivity towards methyl lactate (S(MeLa)), and yield of methyl lactate (Y(MeLa)) for the methanolysis of PLA using guanidine-modified microgels as catalyst under inert conditions.[a] Onefold determinations of single experiments are displayed. Average values as threefold determination with standard deviation are displayed in Table 1 in the main publication.

entry	catalyst	experiment	*X*(int) [%]	*S*(MeLa) [%]	*Y*(MeLa) [%]
**1**	B-TMGtegma+ 5 mol%	1	100	98	98
**2**		2	100	91	91
**3**		3	99	89	88
**4**	B-TMGtegma+ 10 mol%	1	99	93	92
**5**		2	99	91	90
**6**		3	100	89	89
**7**	B-TMGtegma+ 15 mol%	1	99	79	79
**8**		2	99	88	87
**9**		3	97	86	84
**10**	SB-TMGtegma+ 5 mol%	1	98	77	75
**11**		2	96	78	75
**12**		3	100	83	83
**13**	SB-TMGtegma+ 10 mol%	1	96	63	61
**14**		2	95	63	60
**15**		3	99	60	59
**16**	SB-TMGtegma+ 15 mol%	1	96	46	44
**17**		2	99	57	56
**18**		3	99	55	55
**19**	TMGtegma+	1	8	33	2
**20**		2	6	36	2
**21**		3	7	46	3

[a] Standard procedure: screw cap Schlenk tube, N_2 atmosphere, 110 °C, 6 h, 260 rpm, 0.50 mol% catalyst loading (regarding the polymer ester bond in PLA and corresponding to the guanidine units within the microgel), MeOH (2.00 mL, 14.2 equiv), PLA (250 mg, 1.00 equiv, bio-mi Ltd.).

Table S9. Conversion of PLA (X(int)), selectivity towards methyl lactate (S(MeLa)), and yield of methyl lactate (Y(MeLa)) for the methanolysis of PLA using guanidine-modified microgels as catalyst under aerobic conditions.[a] Onefold determinations of single experiments are displayed. Average values as threefold determination with standard deviation are displayed in Table 1 in the main publication.

entry	catalyst	experiment	X(int) [%]	S(MeLa) [%]	Y(MeLa) [%]
**1**		1	98	86	85
**2**	B-TMGtegma+ 5 mol%	2	98	81	79
**3**		3	97	57	55
**4**		1	98	81	79
**5**	B-TMGtegma+ 15 mol%	2	100	70	70
**6**		3	97	58	56
**7**		1	97	68	66
**8**	SB-TMGtegma+ 5 mol%	2	99	56	56
**9**		3	99	69	68
**10**		1	99	52	52
**11**	SB-TMGtegma+ 15 mol%	2	98	42	41
**12**		3	99	50	49
**13**		1	7	38	3
**14**	TMGtegma+	2	14	35	5
**15**		3	18	42	7

[a] Standard procedure: screw cap Schlenk tube, air atmosphere, 110 °C, 6 h, 260 rpm, 0.50 mol% catalyst loading (regarding the polymer ester bond in PLA and corresponding to the guanidine units within the microgel), MeOH (2.00 mL, 14.2 equiv), PLA (250 mg, 1.00 equiv, bio-mi Ltd.).

Recycling of the Guanidine-Modified Microgels under Aerobic Conditions

Table S10. Conversion of PLA (*X*(int)), selectivity towards methyl lactate (*S*(MeLa)), and yield of methyl lactate (*Y*(MeLa)) for the repeatedly performed methanolysis of PLA using B-TMGtegma+ 5 mol% as catalyst under aerobic conditions recycling the catalyst after each reaction cycle.[a] Onefold determinations of single experiments are displayed.

entry	experiment	cycle	*X*(int) [%]	*S*(MeLa) [%]	*Y*(MeLa) [%]
**1**	1	1	98	86	85
**2**		2	68	29	19
**3**		3	64	15	9
**4**		4	55	22	12
**5**	2	1	98	81	79
**6**		2	91	36	33
**7**		3	77	25	20
**8**		4	62	20	12
**9**	3	1	97	57	55
**10**		2	86	32	27
**11**		3	73	23	17
**12**		4	70	17	12

[a] Standard procedure: screw cap Schlenk tube, air atmosphere, 110 °C, 6 h, 260 rpm, 0.50 mol% catalyst loading (regarding the polymer ester bond in PLA and corresponding to the guanidine units within the microgel), MeOH (2.00 mL, 14.2 equiv), PLA (250 mg, 1.00 equiv, bio-mi Ltd.); after the reaction, volatile components were removed under reduced pressure and the reaction vessel was recharged with MeOH (2.00 mL, 14.2 equiv) and PLA (250 mg, 1.00 equiv, bio-mi Ltd.) for a new reaction cycle.

Table S11. Conversion of PLA (X(int)), selectivity towards methyl lactate (S(MeLa)), and yield of methyl lactate (Y(MeLa)) for the repeatedly performed methanolysis of PLA using B-TMGtegma+ 15 mol% as catalyst under aerobic conditions recycling the catalyst after each reaction cycle.[a] Onefold determinations of single experiments are displayed.

entry	experiment	cycle	X(int) [%]	S(MeLa) [%]	Y(MeLa) [%]
**1**	1	1	97	58	56
**2**		2	82	34	28
**3**		3	65	23	15
**4**		4	71	15	11
**5**	2	1	94	57	54
**6**		2	97	34	33
**7**		3	84	31	26
**8**		4	87	22	19
**9**	3[b]	1	100	70	70
**10**		2	62	20	13
**11**		3	n.d.	n.d.	n.d.
**12**		4	n.d.	n.d.	n.d.

[a] Standard procedure: screw cap Schlenk tube, air atmosphere, 110 °C, 6 h, 260 rpm, 0.50 mol% catalyst loading (regarding the polymer ester bond in PLA and corresponding to the guanidine units within the microgel), MeOH (2.00 mL, 14.2 equiv), PLA (250 mg, 1.00 equiv, bio-mi Ltd.); after the reaction, volatile components were removed under reduced pressure and the reaction vessel was recharged with MeOH (2.00 mL, 14.2 equiv) and PLA (250 mg, 1.00 equiv, bio-mi Ltd.) for a new reaction cycle. [b] Recycling experiment had to be discarded after the second reaction cycle due to issues during the work-up.

Table S12. Conversion of PLA (X(int)), selectivity towards methyl lactate (S(MeLa)), and yield of methyl lactate (Y(MeLa)) for the repeatedly performed methanolysis of PLA using SB-TMGtegma+ 5 mol% as catalyst under aerobic conditions recycling the catalyst after each reaction cycle.[a] Onefold determinations of single experiments are displayed.

entry	experiment	cycle	*X*(int) [%]	*S*(MeLa) [%]	*Y*(MeLa) [%]
**1**	1	1	97	68	66
**2**		2	89	41	37
**3**		3	74	23	17
**4**		4	69	26	18
**5**	2	1	99	56	56
**6**		2	87	27	24
**7**		3	77	35	27
**8**		4	68	19	13
**9**	3	1	99	69	68
**10**		2	91	31	28
**11**		3	80	34	27
**12**		4	68	25	17

[a] Standard procedure: screw cap Schlenk tube, air atmosphere, 110 °C, 6 h, 260 rpm, 0.50 mol% catalyst loading (regarding the polymer ester bond in PLA and corresponding to the guanidine units within the microgel), MeOH (2.00 mL, 14.2 equiv), PLA (250 mg, 1.00 equiv, bio-mi Ltd.); after the reaction, volatile components were removed under reduced pressure and the reaction vessel was recharged with MeOH (2.00 mL, 14.2 equiv) and PLA (250 mg, 1.00 equiv, bio-mi Ltd.) for a new reaction cycle.

Table S13. Conversion of PLA (X(int)), selectivity towards methyl lactate (S(MeLa)), and yield of methyl lactate (Y(MeLa)) for the repeatedly performed methanolysis of PLA using SB-TMGtegma+ 15 mol% as catalyst under aerobic conditions recycling the catalyst after each reaction cycle.[a] Onefold determinations of single experiments are displayed.

entry	experiment	cycle	X(int) [%]	S(MeLa) [%]	Y(MeLa) [%]
**1**	1	1	97	42	41
**2**		2	90	42	38
**3**		3	82	31	25
**4**		4	84	38	32
**5**	2	1	98	42	41
**6**		2	76	31	24
**7**		3	84	32	27
**8**		4	76	30	23
**9**	3	1	99	50	49
**10**		2	100	45	45
**11**		3	84	19	16
**12**		4	76	25	19

[a] Standard procedure: screw cap Schlenk tube, air atmosphere, 110 °C, 6 h, 260 rpm, 0.50 mol% catalyst loading (regarding the polymer ester bond in PLA and corresponding to the guanidine units within the microgel), MeOH (2.00 mL, 14.2 equiv), PLA (250 mg, 1.00 equiv, bio-mi Ltd.); after the reaction, volatile components were removed under reduced pressure and the reaction vessel was recharged with MeOH (2.00 mL, 14.2 equiv) and PLA (250 mg, 1.00 equiv, bio-mi Ltd.) for a new reaction cycle.

Table S14. Conversion of PLA (*X*(int)), selectivity towards methyl lactate (*S*(MeLa)), and yield of methyl lactate (*Y*(MeLa)) for the repeatedly performed methanolysis of PLA using TMGtegma+ as catalyst under aerobic conditions recycling the catalyst after each reaction cycle.[a] Onefold determinations of single experiments are displayed.

entry	experiment	cycle	*X*(int) [%]	*S*(MeLa) [%]	*Y*(MeLa) [%]
**1**	1	1	7	38	3
**2**		2	15	11	2
**3**		3	67	23	16
**4**		4	90	25	23
**5**	2	1	14	35	5
**6**		2	13	14	2
**7**		3	33	16	5
**8**		4	81	33	27
**9**	3	1	18	42	7
**10**		2	16	12	2
**11**		3	37	14	5
**12**		4	66	23	15

[a] Standard procedure: screw cap Schlenk tube, air atmosphere, 110 °C, 6 h, 260 rpm, 0.50 mol% catalyst loading (regarding the polymer ester bond in PLA), MeOH (2.00 mL, 14.2 equiv), PLA (250 mg, 1.00 equiv, bio-mi Ltd.); after the reaction, volatile components were removed under reduced pressure and the reaction vessel was recharged with MeOH (2.00 mL, 14.2 equiv) and PLA (250 mg, 1.00 equiv, bio-mi Ltd.) for a new reaction cycle.

References

1 H. Wittmann, V. Raab, A. Schorm, J. Plackmeyer and J. Sundermeyer, *Eur. J. Inorg. Chem.*, 2001, **2001**, 1937–1948.

2 S. Herres-Pawlis, A. Neuba, O. Seewald, T. Seshadri, H. Egold, U. Flörke and G. Henkel, *European J. Org. Chem.*, 2005, **2005**, 4879–4890.

3 P. McKeown, L. A. Román-Ramírez, S. Bates, J. Wood and M. D. Jones, *ChemSusChem*, 2019, **12**, 5233–5238.

4 L. A. Román-Ramírez, P. McKeown, M. D. Jones and J. Wood, *ACS Catal.*, 2019, **9**, 409–416.

5 L. Burkart, A. Eith, A. Hoffmann and S. Herres-Pawlis, *Chem. - An Asian J.*, 2023, **18**, 1–7.

6 H. U. Zaman, J. C. Song, L. S. Park, I. K. Kang, S. Y. Park, G. Kwak, B. S. Park and K. B. Yoon, *Polym. Bull.*, 2011, **67**, 187–198.

7 X. Shi, G. Zhang, C. Siligardi, G. Ori and A. Lazzeri, *J. Nanomater.*, 2015, **2015**, 1–11.

8 X. Wang, J. Mi, J. Wang, H. Zhou and X. Wang, *RSC Adv.*, 2018, **8**, 34418–34427.

9 Y. Luo, Z. Lin and G. Guo, *Nanoscale Res. Lett.*, 2019, **14**, 56.
